# The doMESTIC RISK Tool: Prioritising Home-Care Patients for Clinical Pharmacy Services with the Help of a Delphi Study

**DOI:** 10.3390/nursrep15050158

**Published:** 2025-05-01

**Authors:** Carla Meyer-Massetti, Stefanie Lettieri-Amstutz, Daniela Rölli, Christoph R. Meier

**Affiliations:** 1 Clinical Pharmacy & Epidemiology, Department of Pharmaceutical Sciences, University of Basel, 4056 Basel, Switzerlandchristoph.meier@unibas.ch (C.R.M.); 2 Institute of Primary Health Care (BIHAM), University of Bern, 3012 Bern, Switzerland; 3 Hospital Pharmacy, University Hospital of Basel, 4056 Basel, Switzerland

**Keywords:** home care, medication-related problems, medication safety, risk factors, risk assessment, clinical pharmacy, interprofessional collaboration

## Abstract

**Background:** Medication-related problems (MRPs) are common among home-care patients urgently needing improvement. Due to limited resources, patients with the greatest risk of MRPs should be prioritised for interventions. **Objectives:** We sought to develop a collaborative assessment tool for nurses and pharmacists to identify home-care patients at high risk of developing MRPs. **Methods:** Using Pubmed^®^ for a first scoping literature review, we sought existing tools identifying patients at risk of MRPs or prioritising patients for clinical pharmacy services. Extracted items were prioritised in a first interprofessional Delphi round. Results from the first Delphi round were complemented by individual risk factors identified in a second scoping literature review and again submitted to the expert panel in a second Delphi round. Each item was rated for its relevance to identify home-care patients at risk of MRPs. The highest scoring factors were combined into an interprofessional assessment tool. **Results:** Literature review one yielded 19 risk tools and six lists containing potentially inappropriate medications. The 78 resulting risk factors were submitted to experts (five pharmacists, five physicians, five nurses) in Delphi round one. Since the identified tools did not fit the scope (interprofessional assessment in home care) entirely, the results of Delphi round one were complemented with individual risk factors identified in a second literature review, leading to 82 potential risk factors for Delphi round two. Experts decided on a 15-item tool for future pilot testing—the doMESTIC RISK tool. It incorporated diverse factors potentially influencing medication safety: demographic information, social context, diagnosis, specific medication and health care resources use. **Conclusions:** With expert feedback from a Delphi process, we developed a 15-item tool to help nurses and pharmacists jointly identify home-care patients at a high risk of MRPs. Validation of the doMESTIC RISK tool will be imperative to verify its value in clinical practice.

## 1. Introduction

The patients cared for by professional home-care organisations are predominantly multimorbid, polymedicated older adults and, thus, a population especially vulnerable to medication-related problems (MRPs), including adverse drug reactions and medication errors [1]. Care interfaces, where different professionals work together and transition between different care settings (e.g., ambulatory, inpatient and long-term care), are highly prevalent in home-care settings. This can challenge the interprofessional availability of timely, comprehensive and accurate medication-related information [2]. It is not surprising that previous studies have found MRPs to be far more common among this population [3].

However, studies have also shown that MRPs are often avoidable [4]. Clinical pharmacy services, consisting among others of medication reconciliation at care transitions and medication reviews [5], are well established in inpatient settings in general and at hospital discharge specifically [6]. Clinical pharmacy can positively influence economic, clinical and patient safety outcomes [7,8]. Its use is still limited in the ambulatory-care sector, where multiple care interfaces can hamper the sharing of and access to information relevant for medication reconciliation and analysis and where interprofessional communication is more difficult due to recurring care transition situations [2,3,9].

Not only will the demographic development challenge the health care system, but also the shortage of skilled labour [10]. There are indications in the literature that interprofessional collaboration can positively affect efficient provision of health care and patient outcomes [11]. In home care, interprofessional collaboration is still infrequent and the coordination is often in the hands of patients of informal caregivers [12]. Schmitz et al. reported that common assessment tools can facilitate interprofessional collaboration and communication between health care professionals [13]. Therefore, tools applied in the home health-care sector should facilitate interprofessional collaboration, for example, through joint assessment and sharing of pertinent clinical information.

If service availability is limited, the prioritisation of patients might also be a valuable approach to manage the workload and provide service to patients most in need [14].

### Aims

Based on risk factors specific to home-care populations, the present study aimed to develop an assessment tool to identify vulnerable older home-care patients at a high risk of MRPs and to prioritise them for clinical pharmacy services. A prerequisite for this was incorporating items that would facilitate interprofessional collaboration between home-care nurses and clinical pharmacists.

## 2. Materials and Methods

We performed a two-round Delphi study in Switzerland based on the two-round RAND/UCLA Delphi panel method (www.rand.org) (accessed on 23 February 2025), following the guidance on Conducting and REporting DElphi Studies (CREDES) [15]. Two complimentary scoping literature reviews informed the Delphi rounds before the research team consolidated the risk factors. (1) A first scoping review for the identification of pre-existing tools to identify or prioritise patients for clinical pharmacy services informed (2) the first Delphi round. As none of the tools were an ideal fit for our setting—targeting home-care patients and offering an interprofessional approach—(3) a second literature review for the identification of additional individual risk factors was incorporated in the preparation for (4) Delphi round two.

**Figure 1:**
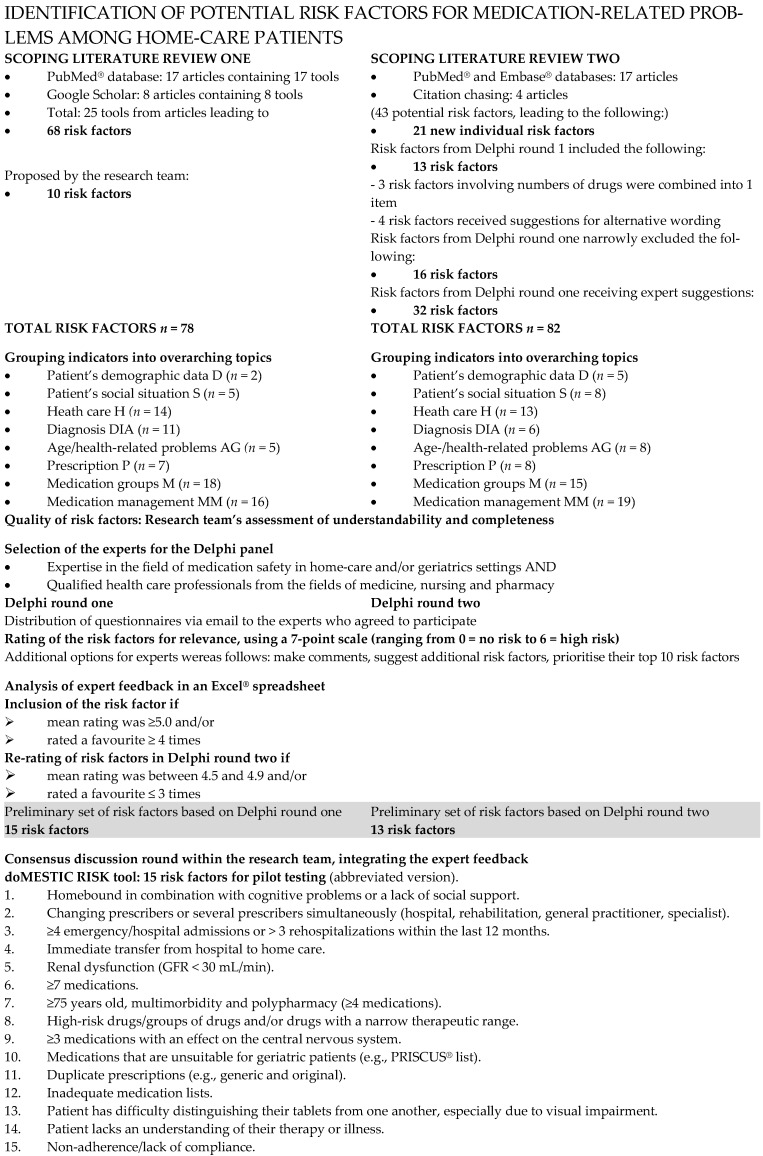
Delphi consensus process.

Well-structured data on home-care settings are limited in the current literature in general; therefore, some risk factors may have been under-represented in the literature, which justified our methodological approach in the format of this Delphi study, giving a voice to experts in the field. A detailed overview of the Delphi process is shown in Figure 1.

### 2.1. Scoping Literature Review One—Identifying Existing Tools and the Risk Factors Covered

Guided by Arksey et al.’s recommendations [16], we undertook a scoping literature review in the PubMed^®^ database to compile risk assessment items for our first Delphi round.

We sought tools used by other institutions to identify patients at a high risk of MRPs or to prioritise patients for receiving clinical pharmacy services. Four topic groups were used to guide the search and extract relevant items: (1) medication safety, (2) risk factors, (3) older adult patients and (4) tools. The search strategy’s details are shown in Supplementary Material S1. The PubMed^®^ search was complemented with citation chasing. Article titles and abstracts were screened independently by two authors. One author extracted the data, and a second author independently verified them.

Drugs or active ingredients that were unavailable in Switzerland at the time of review, but mentioned in the lists of potentially inappropriate medications (PIMs), were not considered. Risk factors inapplicable in home-care settings, or for which information is generally unavailable in home-care data, were excluded after discussions within the research team.

The risk factors identified through the literature search were assessed for clarity and completeness by the research team.

All the risk factors were then compiled in an Excel^®^ spreadsheet for assessment by our expert group. For more clarity, risk factors were listed under the following categories: demographic data, social situation, health care, diagnoses, age-related or health-related problems, prescription, medications or groups of medications and medication management. For additional information, the references for each factor and the number of mentions in the literature were added to the Excel^®^ spreadsheet. The Delphi material was sent to the experts by email. An English version of the table is available in Supplementary Material S2 (translated using www.deepl.com, Cologne, Germany, 10 December 2024). The original German version is available from the authors upon request.

### 2.2. Delphi Round One

Panellists for the Delphi study were chosen based on their expertise in the field of medication safety in home-care and/or geriatrics settings and their qualification as health care professionals from the fields of medicine, nursing and pharmacy, which we wanted equally represented. The number of nurses consenting to participate defined the final number of five experts per professional group.

By participating in the Delphi study, the experts consented to the use of anonymized data in research and a peer-reviewed publication.

Based on their personal experience and expertise, the interprofessional expert panel’s task was to evaluate the proposed set of risk factors concerning their relevance for identifying patients at a high risk of developing MRPs. To perform this, they scored each item using a 7-point scale (ranging from 0 = no risk to 6 = high risk). The experts were also free to expand the list presented to them with supplementary risk factors and provide their comments. Finally, the Delphi panellists were asked to review the risk factors again and to name the ten risk factors they considered the main indicators of MRPs (their “favourites”).

The panellists’ input was analysed using Excel^®^ software (Microsoft Office Professoinal Plus 2016). Risk factors were included in Delphi round two if their mean score was ≥5.0, and/or they were selected as a favourite factor ≥ 4 times. Risk factors were considered to have narrowly failed selection if they had a mean score between 4.5 and 4.9 and/or were selected as a favourite ≤ 3 times.

### 2.3. Scoping Literature Review—Individual Risk Factors

To expand our set of potential risk factors, a second scoping literature review was performed to identify individual risk factors with the potential to be integrated into a risk assessment tool. This review complemented the first review focusing on existing tools. We used an adapted patient/population, intervention, comparison and outcomes (PICO) process by examining the following topics instead: population (P), service (I^1^), service provider (I^2^) and setting (S).

The search strings, available in Supplementary Material S3, were developed for the PubMed^®^ and Embase^®^ databases and limited to the period from 1 January 1998 to 31 December 2018. Additional inclusion criteria were older adults ≥ 64 (P) and the services provided by (clinical) pharmacists addressing MRPs (I^1^, I^2^) in ambulatory-care settings with a focus on professional home care (S). Titles and abstracts were screened independently by two reviewers. Data were extracted independently by one author and verified by another. Relevant items were tabulated for inclusion in Delphi round two, indicating their origin and how often they were mentioned in the literature. This process also followed the PRISMA-ScR guidelines [17].

### 2.4. Delphi Round Two

The items to assess in the scope of Delphi round two were also presented to the expert panel in an Excel^®^ spreadsheet structured into four sections.

Section 1: Contained the risk factors given the highest relevance ratings in Delphi round one and needing specification.

Section 2: Contained the risk factors that narrowly missed selection in Delphi round one and had to be reassessed in round two.

Section 3: Contained the top ten risk factors selected by the expert panel during Delphi round one.

Section 4: Contained the individual risk factors found in the second literature review.

As in Delphi round one, the material was sent to the experts by email and the panel experts were asked to rate the items presented to them on a 7-point scale (ranging from 0 = no risk to 6 = high risk). In addition, the experts were allowed to provide their preferred wording for specific risk factors in Section 1 and to add their remarks and prioritise the indicators in each group.

Risk factors were included within the scope of Delphi round two if they had a mean rating of ≥5.0 (considerable risk) and/or were named as favourites by at least four Delphi panel participants.

### 2.5. Consensus Round

After completing the Delphi process, the research group consolidated the results from both Delphi rounds into a final tool for a future pilot project. The goal was to keep feasibility in mind and not exceed a maximum of 15 risk factors. For the complete Delphi process see Figure 1.

The study was conducted according to the guidelines of the Declaration of Helsinki, and the Ethics Committee of Central and Northwestern Switzerland approved this project as an integral part of the overarching doMESTIC–Medication Safety in Home Care study (EKNZ 2019-00964).

For writing our manuscript, we applied the Preferred Reporting Items for Systematic reviews and Meta-Analyses extension for Scoping Reviews (PRISMA-ScR) [17].

## 3. Results

### 3.1. Scoping Literature Review—Existing Tools and Their Risk Factor Items

Our literature searches identified 17 tools supplemented by eight tools identified through citation chasing, thus resulting in 25 tools in total. An overview of the identified tools is shown in Supplementary Material S4.

Nineteen of the twenty-five tools were used to identify patients at an increased risk of MRPs or to prioritise them for clinical pharmaceutical services (highlighted in blue in Supplementary Material S4’s literature table) [18,19,20,21,22,23,24,25,26,27,28,29,30,31,32,33,34,35]. The other six were lists from Europe defining PIMs for geriatric patients (highlighted in yellow in Supplementary Material S4’s literature table) [36,37,38,39,40,41]. Among these were the Norwegian General Practice Nursing Home (NORGEP-NH) criteria [36], the EU(7)-PIM list [37], the STOPP/START criteria (2015) [38], the PRISCUS list (2010) [39,40] and the GheoP^3^S-tool [41]. The “indicators of unsafe medication practice” were also assigned to this group, encompassing 18 prescription and four monitoring indicators used in the electronic monitoring of medication safety [42]. The FORTA list [43] was not considered during the scoping literature search because the classification of medications into the four categories (A–D) depends on the indication area. This would require too many clarifications for an efficient initial assessment of whether a medication is a high-risk drug.

In addition to the PIM lists, another nine tools developed in Europe were identified [20,22,26,28,29,30,34,35,44]. These are used in different settings, with six tools that were developed specifically for use with inpatients [22,28,29,30,31,44]. The others are used primarily in outpatient settings, with the Drug-Related Problem Risk Assessment Tool [20] and the Safe Medication Assessment Tool [26] being used in home-care settings. Some tools provide support at care interfaces. The Composite Care Transitions Score is used in transitions from hospital to outpatient settings [24], while the PADR-EC Score [18] and the 80+ Score [22] are used to determine the risks of rehospitalisation due to MRPs.

About half of the 19 tools selected are used specifically with older adults [18,20,22,25,26,27,28,29,31,32], while the other six tools are also used for patients < 65 years old. Nevertheless, some of the latter group of tools cite increased age as a risk factor [24,35].

Many of the risk factors included in these tools had themselves been identified through prior literature reviews [20,24,25,30,31,32,34,35], and the contents of three tools [20,30,31] had subsequently been validated using a consensus method, such as a Delphi process. Other tools took risk factors from existing instruments and adapted them to their respective settings [19,21,23,27], sometimes only selecting risk factors with proven outcomes in previous studies [19,21]. Four studies determined their risk factors via retrospective analyses of data from patients with MRPs [18,22,29,44]. Many studies tested whether their tool’s expected outcomes could be achieved, with some studies considering this to be “internal validation” [18,22,23,29,32,44] and others using the term evaluation [19,25,26,27]. We identified four tools validated separately from studies in the further literature [20,31,45,46]. Barnett et al.’s Prevent Tool [34] and the Risk Indicators for Medicines-related Problems Tool [35] had not been validated at the time of writing.

A set of 78 risk factors was presented to the expert panel (for the original form, see Supplementary Material S6). Of these, two risk factors were listed in the demographic data category, five in the social situation and age- or health-related problems categories, respectively, seven addressed prescribing, 11 were diagnosis-specific risk factors, 14 belonged to the health care category, 16 addressed patients’ medication management, and 18 were categorised as specific medications or medication groups.

### 3.2. Delphi Round One

All 15 of the expert panellists—five physicians, pharmacists and nurse specialists, respectively—invited to participate in the Delphi round one process accepted the invitation, and Table 1 shows their sociodemographic characteristics.

Fifteen of the risk factors presented to the expert panel met our tool inclusion criteria, with ten meeting the required mean score, eleven being considered major indicators of MRPs by four or more Delphi participants, and six risk factors meeting both criteria. The categories most frequently selected for their relevance addressed polypharmacy (P2, P3 and P4) and patients’ medication management difficulties (MM1, MM5, MM10 and MM12). Overall, the experts considered risk factors from a wide range of different domains to be important, as shown in Table 2.

The expert panel also suggested 25 other risk factors; however, these were all variations or specifications of risk factors presented in Delphi round one. They were, therefore, presented as choice items or for specification.

### 3.3. Scoping Literature Review for Individual Risk Factors

Our second scoping literature review (see Supplementary Material S3 for the PRISMA (17) flowchart) yielded 43 additional risk factors suitable for evaluation by our expert panel. These came from an analysis of 21 full texts, 17 of which were found via our search strategy and four of which came from citation. The list of included references as well as the risk factors extracted from these sources are displayed in Supplementary Material S5. Of note, 21 risk factors were new and added to the Delphi round two. They were mentioned between one and nine times, respectively. Risk factors which were mentioned more often were already part of Delphi round one.

### 3.4. Delphi Round Two

The final Excel^®^ spreadsheet for Delphi round two contained 82 risk factors for assessment and/or specification, separated into the four groups as described in the Methods section (see details in Supplementary Material S6 and Figure 1). Delphi round two’s expert panel was identical to round one. They prioritised eight risk factors with a mean rating of ≥5.0 and nine risk factors featuring among the panellists’ favourites ≥ 4 times. Four risk factors had a mean rating ≥ 5.0 and were also favourites: P9, M24, MM9 and MM22. An overview of the 13 risk factors making the cut in Delphi round two is shown in Table 3.

### 3.5. Consensus Round

Based on expert feedback from Delphi survey rounds one and two and internal discussions, the research team optimised the list of risk factors, ending with a set of 15 risk factors for future pilot testing. At the experts’ suggestion, some factors were made more specific or combined into a single factor. While every factor from Delphi round one could be integrated into our risk assessment tool, factors MM23 (inadequate monitoring with irregular follow-up) and P10 (clinically relevant interaction without possible monitoring), prioritised in Delphi round two, were ultimately omitted, despite reaching cut-off levels. This was due to a lack of available information in home-care settings. The set of risk factors, including their origins, for future pilot testing, including their original factors but with adapted wording, is shown in Table 4.

## 4. Discussion

In the context of limited ambulatory-care resources, we sought to create a tool to help health care professionals prioritise which home-care patients should benefit from clinical pharmacy services. The present iterative two-round Delphi process involved an interprofessional panel of experts from different medical specialties and settings and resulted in the development of a final risk assessment tool—the doMESTIC RISK tool—featuring 15 risk factors. The tool includes items requiring data from a range of domains, including demographic information, the social context, diagnosis, specific medication and health care use. The diversity of the factors selected reflects the need to consider multiple parameters when assessing the risk of MRPs [47].

The first scoping literature review revealed that few tools existed for identifying patients at risk of MRPs, and even fewer were specific to home-care settings. The Drug-Related Risk Assessment Tool [20] and the Safe Medication Assessment Tool [26] were the only two found to target this population specifically. However, both these tools are intended for use by nurses exclusively, underscoring the complete lack of collaborative interprofessional tools. Due to the lack of a tool specific to home-care settings, it made sense to reassess the relevance of our extracted items with these settings in mind. This makes our tool unique in the sense that it addresses home-care patients specifically and incorporates interprofessional viewpoints when assessing risk.

The risk factors originating from the tools discovered have been validated to different degrees. With the exception of two tools [34,35], the instruments identified in our scoping literature research were tested for achieving their target outcomes. Various approaches were used to validate, review or evaluate these tools, and different conclusions were drawn (see Supplementary Material S4).

Although our initial search strategy was limited to PubMed^®^, complemented by citation chasing, new items suggested by our panel experts within the scope of the Delphi round one process correlated closely with items already extracted from the literature. This seems to indicate that our approach identified a wide range of risk factors despite the use of a single database.

However, we counteracted this potential limitation by expanding our search to complementary individual risk factors in our second scoping literature review and Delphi round. The second scoping literature review yielded only a limited number of additional risk factors for consideration by the expert panel that were mostly mentioned only a few times in the literature.

Consensus methods like a Delphi process are particularly used when empirical data on a given issue are limited or completely lacking [48,49]. A Delphi process offers the possibility of combining the knowledge and experience of several experts and obtaining the best possible assessment of a specific issue [48].

Two of the tools revealed in our scoping literature review also used a Delphi process to identify risk factors: the Drug-Related Risk Assessment Tool [20] and the Drug-Associated Risk Tool [30]. The weighting of the risk factors in the Assessment of Risk Tools study was also determined using a group consensus method [31]. Lastly, many PIM lists have also been developed based on the consensus of expert panels as part of the Delphi processes [36,37,38,39].

Our interprofessional expert panel was deliberately chosen so that the assessment of the risk factors incorporated different perspectives. This reflected the need for a multimodal interprofessional approach to improving medication safety for the long term, as postulated by the World Health Organization’s Medication Without Harm programme [47]. The literature considers bringing together experts with different backgrounds and knowledge as an important aspect of creating expert consensus. This prevents assessments based on the point of view of one particular group of specialists, which could lead to a one-sided or biased result [49]. We consider our expert panel’s exceptional response rate and consistency of participation across both Delphi rounds to be one of our study’s strengths. Nevertheless, the overall number of participating experts was only five panellists per professional group. While Delphi studies can incorporate various numbers of experts and an optimal size has not been established [48], 15 experts might be on the lower end of the spectrum. Due to difficulties with recruiting nurses considering themselves experts in medication safety, equal representation led to this composition of the expert panel.

Although we invited experts to join our panel based on their diverse professional backgrounds, knowledge of home-care settings and/or expertise in treating older adult populations, there is a risk of selection bias because they were all recruited from the study researchers’, albeit wide, circle of colleagues.

Our two rounds of the Delphi consensus process led to the inclusion of potential risk factors that were rated for inclusion in a tool for prioritising older adult home-care patients with multimorbidity and polypharmacy for clinical pharmacy services. However, in the final consensus discussion, it became apparent that the inclusion of several specific risk factors, although deemed important, lacked feasibility due to the limited availability of the necessary data in home-care settings. A future project could investigate not only the relevance of specific risk factors but also the feasibility of including them.

Ensuring our newly developed tool’s feasibility in clinical practice also meant limiting the number of risk factors integrated into it. Thus, several factors were re-worded or merged based on their thematic correlation. The feasibility of this approach and of the tool itself now needs psychometric validation in a clinical setting. In the scope of this study, we have focused on the relevance of risk factors to assess the risk for medication-related problems. However, the experts did not comment on the feasibility of detecting those risk factors in daily clinical practice. In addition, clarification would be needed for the role allocation between nurses and pharmacists in the application of the tool as well as a definition for a specific cut-off score leading to clinical pharmacy services.

## 5. Conclusions

The present study used a two-round Delphi process with an interprofessional panel of experts to compile an instrument—the doMESTIC RISK tool—for prioritising which home-care patients would benefit most from clinical pharmacy services to improve their medication safety.

This process enabled us to assemble 15 relevant risk factors from diverse domains, reflecting the need to consider the wide range of factors influencing medication safety. Certain risk factors, e.g., polypharmacy, are well established in the literature, while others, addressing a patient’s social context, for instance, are more innovative.

Although the doMESTIC RISK tool aims to promote collaboration between nurses and pharmacists caring for patients in home-care settings and provides a starting point for targeted medication safety interventions, future research should pilot-test this first iteration of the tool and validate the risk factors integrated into it.

## Figures and Tables

**Table 1:** Sociodemographic characteristics of the experts who participated in the Delphi process.

*Characteristic*	*Round 1*	*Round 2*
Experts, n (%)	15 (100%)	15 (100%)
Sex		
* Female, n (%)*	11 (73%)	11 (73%)
* Male, n (%)*	4 (27%)	4 (27%)
Profession		
* Physician, n (%)*	5 (33%)	5 (33%)
* Pharmacist, n (%)*	5 (33%)	5 (33%)
* Nurse, n (%)*	5 (33%)	5 (33%)
Current work setting (multiple options possible)		
* Research, n (%)*	6 (40%)	6 (40%)
* Inpatient care, n (%)*	9 (60%)	9 (60%)
* Home care, n (%)*	4 (27%)	4 (27%)
* Long-term care, n (%)*	1 (7%)	1 (7%)
Medical specialisation (multiple options possible)		
* Clinical pharmacy, n (%)*	5 (33%)	5 (33%)
* Hospital pharmacy, n (%)*	4 (27%)	4 (27%)
* General internal medicine, n (%)*	2 (13%)	2 (13%)
* Geriatrics, n (%)*	3 (20%)	3 (20%)
* Clinical pharmacology, n (%)*	1 (7%)	1 (7%)
* Advanced nurse practitioner, n (%)*	5 (33%)	5 (33%)

**Table 2:** Ranking of the 15 risk factors prioritised in Delphi round one. Values that met our cut-off requirements are highlighted.

N	Topic	Risk Factor	MRFR	SD	Med.	IQR	Min.	Max.	N Favourite Nominations
**1**	**AG5**	Cognitive deficits	**5.4**	0.6	5	1	4	6	**9**
**2**	**P4**	≥8 medications	**5.4**	0.9	6	1	3	6	2
**3**	**MM1**	Patient has no current medication list	**5.2**	0.9	5	1	3	6	**9**
**4**	**MM5**	Patient has difficulty distinguishing medications or has visual difficulties	**5.1**	0.8	5	1.5	4	6	**4**
**5**	**DIA7**	Renal dysfunction (GFR < 30 mL/min)	**5.1**	0.7	5	0.75	4	6	**4**
**6**	**M9**	≥3 psychotropic drugs (centrally acting analgesics, antipsychotics, antidepressants, benzodiazepines)	**5.1**	0.9	5	1.5	3	6	**4**
**7**	**H11**	Hospital admission due to MRPs	**5.1**	1.1	5	1	2	6	2
**8**	**M3**	Medications unsuitable for older adult patients (e.g., PRISCUS^®^ list)	**5.0**	0.8	5	2	4	6	**5**
**9**	**MM12**	Patient lacks an understanding of their therapy and/or illness	**5.0**	0.9	5	0	2	6	2
**10**	**P3**	≥7 medications	**5.0**	1.1	5	2	3	6	0
**11**	**S4**	Communication problems and/or foreign language	4.3	0.9	4	1	3	6	**7**
**12**	**M1**	Medication with narrow therapeutic range	4.8	1.0	5	1.5	2	6	**4**
**13**	**MM10**	Patient takes medication without their doctor’s knowledge (including over-the-counter drugs)	4.8	1.1	5	2	2	6	**4**
**14**	**P2**	≥6 medications	4.5	1.1	4	1.5	2	6	**4**
**15**	**H4**	Frequently changes physician	4.2	1.5	5	1.75	0	6	**4**

MRFR = mean risk factor rating; SD = standard deviation; Med = median; IQR = interquartile range; Min = minimum; Max = maximum; N = number; MRP = medication-related problem. Topics: D = demographic information; DIA = diagnosis; H = health care; M = medication/medication group; MM = medication management; P = prescription; S = social situation.

**Table 3:** The 13 risk factors from Delphi round 2 that met one or both inclusion criteria (mean value and number of favourites). Values that met the cut-off requirements are highlighted.

N°	Abbr.	Risk Factor	MRFR	SD	Med.	IQR	Min.	Max.	N Favourite Nominations
1	P9	≥10 medications	**5.5**	0.6	6	1	4	6	**4**
2	M23	Duplicate prescriptions (e.g., generic and original)	**5.3**	1.2	6	1	1	6	0
3	M24	High-risk drugs/groups of drugs	**5.2**	0.6	5	1	4	6	**7**
4	P10	Clinically relevant interaction with no possible monitoring	**5.1**	0.9	5	1	3	6	1
5	MM2	Poorly comprehensible therapy plan (complex therapy plans)	**5.1**	0.7	5	0.5	4	6	3
6	H23	Several medication lists at the same time, especially if there are several prescribers (various specialists involved)	**5.0**	1.0	5	0.5	2	6	3
7	MM9	Non-adherence/lack of compliance (e.g., incorrect intake due to not understanding the therapy)	**5.0**	1.0	5	1.5	3	6	**4**
8	MM22	Lack of an up-to-date or correct medication list/no medication list	**5.0**	0.6	5	0	4	6	**4**
9	H15	Changing prescribers (hospital, rehabilitation, general practitioner, specialist)	4.7	1.1	5	1.5	2	6	**6**
10	D5	≥75 years old, multimorbidity and polypharmacy	4.7	1.1	5	1.5	3	6	**6**
11	S8	Homebound in combination with cognitive problems or a lack of social support	4.6	1.1	5	1	2	6	**6**
12	DIA13	Neurocognitive disorder of any degree of severity	4.6	1.0	5	1	3	6	**7**
13	MM23	Inadequate monitoring (irregular follow-up)	4.5	0.8	5	1	3	6	**4**

MRFR = mean risk factor rating; SD = standard deviation; Med = median; IQR = interquartile range; Min. = minimum; Max. = maximum; n = number. Topics: D = demographic information; DIA = diagnosis; H = health care; M = medication/medication group; MM = medication management; P = prescription; S = social situation.

**Table 4:** The 15 risk factors of the doMESTIC RISK tool for pilot testing. (Risk factors for which either/or both will be considered during pilot testing are in italics.)

N°	Origin		Risk Factor	Intended Evaluators
	Delphi Round One	Delphi Round Two		
**1**	AG5	S8 DIA13	Homebound in combination with cognitive problems or a lack of social support	Nurse
**2**	H4 (H11)	H15 H23	Changing prescribers or several prescribers simultaneously (hospital, rehabilitation, general practitioner, specialist)	both
**3**	(H11)	new: expert	≥4 emergency/hospital admissions or >3 rehospitalizations within the last 12 months	Nurse
**4**	(H11)	new: expert	Immediate transfer from hospital to home care	Nurse
**5**	DIA7	---	Renal dysfunction (GFR < 30 mL/min)	Pharmacist
**6**	P2 P3 P4	P9	≥7 medications	Pharmacist
**7**	---	D5	≥75 years old, multimorbidity and polypharmacy (≥4 medications)	Both
***8***	*M1*	*M24*	*High-risk drugs/groups of drugs and/or drugs with a narrow therapeutic range* *(neuroleptics, especially lithium, digoxin, amiodarone and other antiarrhythmics; antiepileptics, especially phenytoin; phenobarbital; carbamazepine; oral anticoagulants, especially phenprocoumon; direct oral anticoagulants; insulins; methotrexate; theophylline)*	*Pharmacist*
***9***	*M9*	*---*	*≥3 medications with an effect on the central nervous system* *(centrally acting analgesics; antipsychotics; antidepressants; benzodiazepines)*	*Pharmacist*
**10**	M3	---	Medications unsuitable for older adult patients (e.g., PRISCUS^®^ list)	Pharmacist
**11**	---	M23	Duplicate prescriptions (e.g., generic and original)	Pharmacist
**12**	MM1	MM22	Inadequate medication lists (absence of an up-to-date, understandable or correct medication list or simultaneous existence of several contradictory medication lists)	Both
**13**	MM5	---	Patient has difficulty distinguishing their tablets from one another, especially due to visual impairment	Nurse
**14**	S4 MM12	MM2	Patient lacks an understanding of their therapy and/or illness (cognition and/or communication problems, e.g., foreign language, hearing impairment)	Nurse
**15**	MM10	MM9	Non-adherence/lack of compliance (e.g., incorrect intake due to not understanding the therapy) and/or patient takes medication without the doctor’s knowledge (including self-purchased medication)	Nurse

Topics: AG = age- and health-related problems; D = demographic information; DIA = diagnosis; H = health care; M = medication/medication group; MM = medication management; P = prescription; S = social situation. PRISCUS^®^ list: www.priscus2-0.de (accessed on 23 February 2025).

## Data Availability

Original questionnaires in German are available from the authors upon request.

## Supplementary Materials

The following supporting information can be downloaded at https://www.mdpi.com/article/10.3390/nursrep15050158/s1. Supplementary Material S1 search strategy and PRISMA flow chart (17) for scoping literature review one: risk assessment tools; Figure S1.1: PRISMA 2020 flow diagram. Supplementary Material S2 form for Delphi round one. Supplementary Material S3 search strategy and PRISMA flow chart (17) for scoping literature review two: individual risk factors; Figure S2.1: PRISMA 2020 flow diagram. Supplementary Material S4 overview of the 25 risk assessment factors for medication-related problems identified through literature review one. Supplementary Material S5 overview of the risk factors identified through literature review two; Table S5.1: Risk factors identified by literature review; Table S5.2: References of the risk factors identified by the literature search 2 (in alphabetical order); Table S5.3: Summary of the studies included in literature review 2. Supplementary Material S6 form for Delphi round two.

## Guidelines and Standards Statement

For writing our manuscript, we applied the Preferred Reporting Items for Systematic reviews and Meta-Analyses extension for Scoping Reviews (PRISMA-ScR) [17].
