# Supplementary material for: The doMESTIC RISK Tool: Prioritising Home-Care Patients for Clinical Pharmacy Services with the Help of a Delphi Study
Source: Nurs Rep. 2025 May 1;15(5):158. doi: 10.3390/nursrep15050158 (PMC12114156; doi:10.3390/nursrep15050158)
Supplement: Supplementary file 1 [file nursrep-15-00158-s001.zip › nursrep-3522648-supplementary.pdf]

Supplementary Material S1

Search strategy and PRISMA flow chart for scoping literature review one: risk assessment tools

Research question

What tools exist in order to identify elderly patients at risk for medication-related problems or prioritize patients for clinical pharmacy services?

Population	Elderly patients
Intervention	Tools, risk factors
Outcome	Medication safety, medication-related problems

Eligibility criteria

Inclusion criteria

- Original article
- Population: Patients 64 years and older
- Setting: ambulatory and inpatient care
- Intervention: Tools and risk factors identifying medication-related problems
- Timeframe: January 1, 2000 until January 31, 2018
- Languages: English, German, French

Exclusion criteria

- Review articles, conference abstracts and proceeding papers, editorials, letters and newspaper articles
- Tools addressing specific illnesses, medications, medication classes
- Tools not suitable for the home care setting (e.g., due to missing information)
- Tools for palliative care
- Publications with tools / PIM lists with a more recent version available
- PIM lists without an European version
- Medications not available on the Swiss Market
- Languages other than English, German, French
- Publications before January 1, 2000

Final Search string Pubmed

("Medication Errors"[Mesh] OR "Medication Reconciliation"[Mesh] OR "Inappropriate Prescribing"[Mesh] OR "Drug Prescriptions"[Mesh] OR "Drug-Related Side Effects and

Adverse Reactions"[Mesh] OR “Medication Therapy Management”[Mesh] OR Medication Error[tiab] OR Medication Errors[tiab] OR Medication reconciliation[tiab] OR inappropriate medication[tiab] OR inappropriate drug*[tiab] OR adverse reaction[tiab] OR adverse reactions[tiab] OR medication therapy management[tiab])
AND ("Potentially Inappropriate Medication List"[Mesh] OR "Risk Assessment"[Mesh] OR Tool*[ti] OR Checklist[ti] OR Checklists[ti] OR Questionnaire[ti] OR Questionnaires[ti] OR Score*[ti] OR Assessment[ti] OR Rating[ti] OR Screen*[ti] OR Identification[ti] OR Index[ti] OR Indices[ti] OR criteria[ti] OR classification[ti] OR Predicting[ti] OR detecting[ti] OR detection[ti])
AND ("Risk Factors"[Mesh] OR "Risk Management/methods"[Mesh] OR Indicator[tiab] OR Indicators[tiab] OR high-risk[tiab] OR risk[tiab] OR risks[tiab] OR Trigger*[tiab])
AND ("Aged"[Mesh] OR elderly[tiab] OR elder[tiab] OR elders[tiab] OR old[tiab] OR older adults[tiab] OR geriatric[tiab] OR geriatrics[tiab] OR Aging[tiab])
NOT ("animals"[Mesh] NOT "humans"[Mesh])
NOT (letter [pt] OR newspaper article [pt])
AND ("2000/01/01"[PDAT] : "2018/01/31"[PDAT])

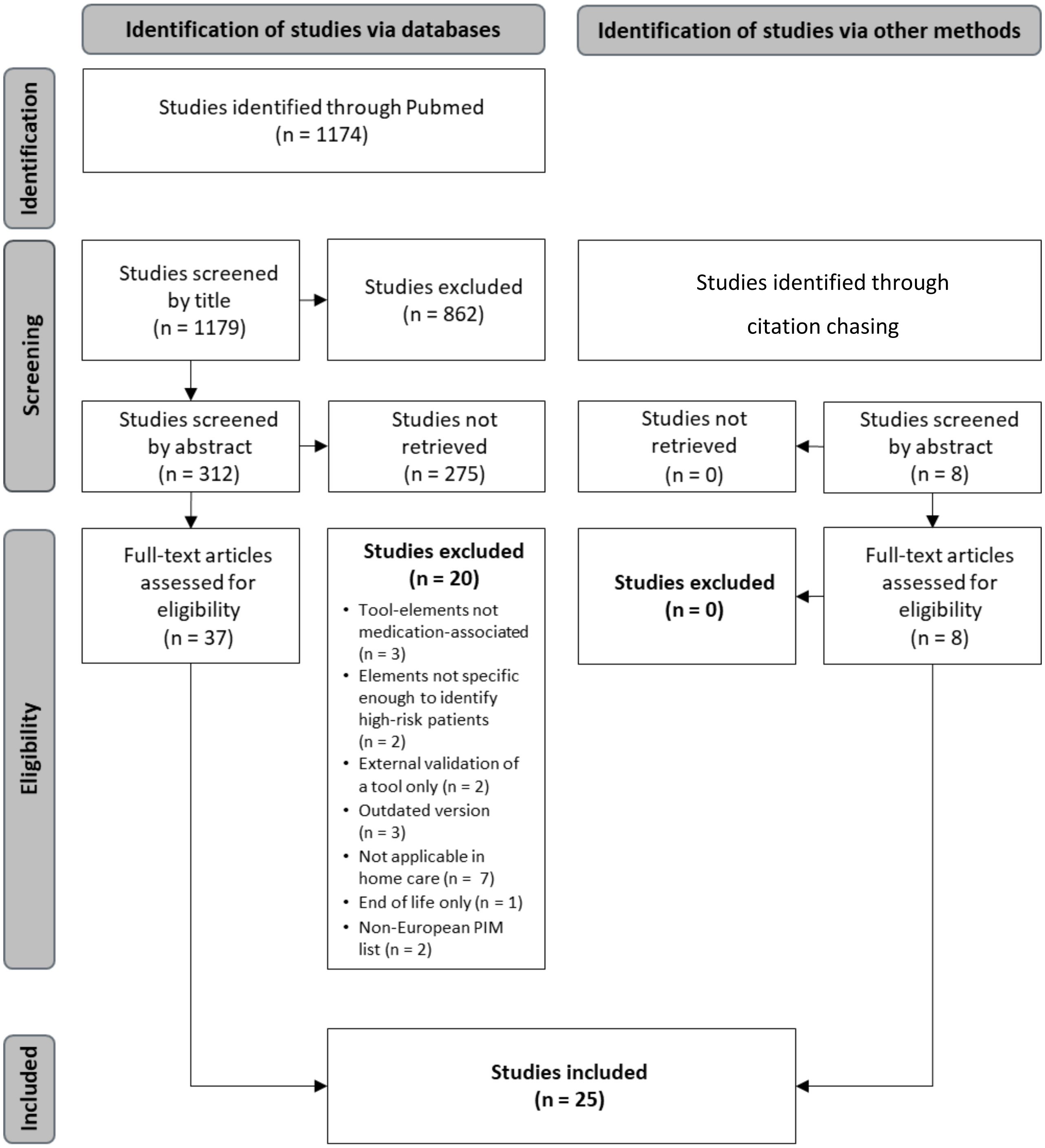

Figure S1.1: PRISMA 2020 [1] flow diagram.

Abbreviations: n, number

1. Tricco AC, Lillie E, Zarin W, O'Brien KK, Colquhoun H, Levac D, Moher D, Peters MDJ, Horsley T, Weeks L, et al. PRISMA Extension for Scoping Reviews (PRISMA-ScR): Checklist and Explanation. Ann Intern Med. 2018;169(7):467–73

Supplementary Material S2: Form Delphi round 1 with all risk factors

Risk assessment - medication-associated problems (DRPs) in Spitex patients.

0=no risk; 1=minimal risk; 2=small risk; 3=medium risk; 4=increased risk; 5=considerable risk; 6=large risk

Name, first name ***(please overwrite)***

		Risk	Nominations Literature	Sources	Comments
	**Patient - demographic data**				
	What age do you consider a risk factor for DRPs?		16	1, 2, 4, 6, 7, 9, 10, 11, 12, 13, 14, 16, 17, 23,	
D1	>65 years		2	9, 23	
D2	>75 years		1	17	
	own proposal (please overwrite)				
	**Patient - social situation**				
	What social situation do you consider a risk factor for DRPs?				
S1	Living alone		2	4, 11	
S2	housebound		1	22	
S3	limited mobility		2	14, 22	
S4	Communication problems - foreign language		2	15, 17	
S5	Communication problems - Hearing problems		*new*		
	own proposal (please overwrite)				
	**Healthcare**				
	Which of the following issues do you consider a risk factor for DRPs?				
H1	No designated family physician		1	17	
H2	more than 1 attending physician		1	4	
H3	Prescriptions from >2 prescribing institutions		1	11	
H4	Frequent change of doctor		1	21	
	own proposal (please overwrite)				
H5	poor acceptance of Spitex services		*new*		
H6	fewer services than Spitex would consider good		*new*		
	own proposal (please overwrite)				
H7	Transfer from hospital to Spitex		1	21	
H8	last hospital admission via emergency		2	12, 21	
H9	unplanned hospital stay within the last 6 months		1	18	
H10	>4 emergency/hospital admissions within the last 12 months		1	17	
H11	Hospital admission due to medication-associated problem		1	22	
H12	≥3 rehospitalizations within one year		1	9	
H13	Rehospitalization <30 days		3	9, 17, 22	
H14	Short-term care (hospital, home, cure) in the last 4 weeks		1	4	
	own proposal (please overwrite)				
	**Diagnoses**				
	What number of chronic conditions do you consider at risk for DRPs?		5	3, 4, 5, 16, 18	
DIA1	Number of chronic diseases ≥3		1	4	
DIA2	Number of chronic diseases ≥4		1	16	
DIA3	Number of chronic diseases with medication use ≥3		3	3, 4, 5	
	own proposal (please overwrite)				
	What diagnoses do you consider to be at risk for DRPs?				
DIA4	Heart failure		5	9, 14, 15, 16, 17	
DIA5	Arrhythmias		7	4, 6, 15, 20, 24	
DIA6	Renal dysfunction GFR <50ml/min		6	1, 7, 14, 15, 16, 23	
DIA7	Renal dysfunction GFR <30ml/min		2	7, 17	
DIA8	Poorly controlled blood pressure		*new*		
DIA9	poorly controlled blood glucose (HbA1c, hypoglycemias)		*new*		
DIA10	Asthma		2	5, 15	
DIA11	COPD		4	5, 14, 15, 17	
	own proposal (please overwrite)				
	**Age/health-related problems**				
	Which of the following issues do you consider a risk to DRPs?				
AG1	Alcohol Abuse		2	11, 22	
AG2	Sleep problems		1	14	
AG3	>1 fall in past 12 months		1	4	
AG4	Fall in past 3 months		1	14	
AG5	Cognitive deficits		5	1, 9, 14, 15, 22	
	own proposal (please overwrite)				
	**Prescription**				
	What number of prescribed medications/medication changes do you consider to be at risk for DRPs?				
P1	Number of drugs ≥5		4	3, 5, 16, 25	
P2	Number of drugs ≥6		4	14, 15, 21, 23	
P3	Number of drugs ≥7		1	4	
P4	Number of drugs ≥8		2	16, 19	
P5	≥4 medication changes in the past year.		1	3	
P6	≥5 medication changes in the past year		1	5	
	own proposal (please overwrite)				

		Risk	Nominations Literature	Sources	Comments
	What situation related to interactions do you consider a risk for DRPs?				
P7	at least 1 interaction with clinical relevance (level 2 according to compendium)		*new*		
	own proposal (please overwrite)				
	What drug/medication groups do you consider to be at risk for DRPs?				
M1	Drugs with narrow therapeutic range		8	3, 4, 6, 9, 12, 15, 24, 25	
M2	Drugs for which monitoring is necessary		3	4, 17, 22	
M3	Medications that are unsuitable for geriatric patients (e.g.Priscus).		>10	13, 24	
M4	Duplicate prescription of a class of drugs		1	13	
M5	Anticoagulants		27	3, 4, 6, 9, 14, 15, 17, 20, 22, 23, 24, 25	
M6	Anti-dementia drugs		8	1, 6, 9, 14, 15, 24, 25	
M7	Antiepileptic drugs		13	3, 4, 6, 12, 15, 17, 24, 25	
M8	Antipsychotics / antidepressants		36	2, 3, 4, 5, 6, 9, 13, 14, 15, 17, 22, 23, 24	
M9	Psychotropic drugs ≥3 (centrally acting analgesics, antipsychotics, antidepressants, benzodiazepines).		1	2	
M10	Benzodiazepines / Z-Drugs		41	2, 6, 13, 24, 25	
M11	Anticholinergics		11	1, 6, 15, 24	
M12	Opioids		15	3, 5, 6, 7, 9, 15, 17, 22, 24, 25	
M13	Opioids without consideration WHO staging scheme		*new*		
M14	Non-steroidal anti-inflammatory drugs		19	2, 3, 4, 5, 6, 14, 15, 22, 23, 24, 25	
M15	Proton pump blocker		3	6, 7, 14	
M16	Antidiabetics - oral		14	5, 6, 9, 14, 15,17, 19, 22, 23, 25	
M17	Antidiabetics - Insulin		11	3, 5, 6, 9, 14, 15, 17, 19, 22, 23	
M18	Metothrexate		5	3, 4, 20, 22, 23	
	own proposal (please overwrite)				
	own proposal (please overwrite)				
	own proposal (please overwrite)				
	own proposal (please overwrite)				
	**Medication Management**				
	Which of the following situations do you consider a risk to DRPs?				
MM1	Patient does not have a current medication list		1	4	
MM2	poorly understandable therapy plan		2	11, 22	
MM3	Medications with difficult handling (e.g. inhalatives)		3	4, 15, 22	
MM4	Patient has poor dexterity (e.g., removal from dispenser)		5	4, 11, 15, 22, 23	
MM5	Patient has difficulty distinguishing the tablets, also visual difficulties		2	15, 22	
MM6	Patient has difficulty swallowing the tablets		5	11, 14, 15, 22, 23	
MM7	Chaotic drug storage		*new*		
MM8	Patient does more himself than Spitex finds useful		*new*		
MM9	Non-adherence in general (poor compliance)		7	4, 10, 15, 17, 18, 22, 25	
MM10	Patient takes medication without doctor's knowledge (also self-purchased)		5	4, 10, 11, 15, 25	
MM11	Patient takes medication differently than prescribed by the doctor		5	4, 8, 10, 11, 22	
MM12	Patient - lack of understanding of therapy and disease (also cognition).		4	4, 10, 11, 15	
MM13	Concerns about medication - patient		5	4, 8, 10, 11, 15	
MM14	Concerns about medication - relatives		1	4	
MM15	Concerns about medication - nurse practitioners		*new*		
MM16	Adverse drug reaction(s) in the past		2	16, 23	
	own proposal (please overwrite)				

	**Favorites**
	Please list MAXIMUM 10 influencing factors (e.g., from the table above) that you consider to be key cues for subsequent DRPs
1	
2	
3	
4	
5	
6	
7	
8	
9	
10	

consider

Supplementary Material S3

Search strategy and PRISMA flow chart for scoping literature review two: individual risk factors

Research question

What risk factors exist in order to prioritize home care patients for clinical pharmacy services?

Population	Home care
Service	Medication safety-related services
Service provider	(Clinical) pharmacist(s)
Setting	Ambulatory care – professional home care

Eligibility criteria

Inclusion criteria

- Population: Patients 64 years and older
- Setting: ambulatory care
- Intervention: Services provided by clinical pharmacy
- Timeframe: January 1, 1998 until December 31, 2019
- Languages: English, German, French, Italian, Spanish

Exclusion criteria

- Population: Patients younger than 64 years
- Languages other than English, German, French, Italian, Spanish
- Publications before January 1, 1998

Final Search string

For the scoping review, Mesh terms and text words were used for the individual search blocks population, setting and intervention and these were combined in a search string. This was done for the following databases: Pubmed and EMBASE.

Search string Pub med

(old-patient[tiab] OR aged[tiab] OR elderly[tiab] OR geriatric[tiab] OR senior[tiab] OR above-65-year-olds[tiab] OR "Aged"[Mesh]) AND (home-care[tiab] OR skilled-care[tiab] OR domiciliary-care[tiab] OR home-health-care[tiab] OR formal-care[tiab] OR home-nursing[tiab] OR nursing-care[tiab] OR home-health-nursing[tiab] OR “Health Services for the

Aged"[Mesh] OR "Home Care Services"[Mesh] OR "Home Nursing"[Mesh] OR "Home Health Nursing"[Mesh] OR "Home Care Agencies"[Mesh] OR "Nursing Services"[Mesh]) AND (medication-management[tiab] OR medication-reconciliation[tiab] OR medical-analysis[tiab] OR pharmaceutical-intervention[tiab] OR pharmaceutical-service[tiab] OR medication-regimen[tiab] OR therapy-management[tiab] OR medication-review[tiab] OR pharmacist-recommendations[tiab] OR drug-utilization-review[tiab] OR drug-management[tiab] OR medication-safety[tiab] OR drug-analysis[tiab] OR prescription-plan[tiab] OR medication-plan[tiab] OR drug-review[tiab] OR brown-bag[tiab] OR “Medication Reconciliation"[Mesh] OR "Medication Therapy Management"[Mesh] OR "Pharmaceutical Services"[Mesh] OR "Risk Assessment"[Mesh] OR "Drug Utilization Review"[Mesh]) AND (pharmacist[tiab] OR Pharmac*[tiab] OR Interdisciplinary-health-teams[tiab] OR "Pharmacists"[Mesh])

<u>Search string EMBASE</u>

(old-patient:ti,ab OR aged:ti,ab OR elderly:ti,ab OR geriatric:ti,ab OR senior:ti,ab OR above-65-year-olds:ti,ab OR 'aged'/exp) AND (home-care:ti,ab OR skilled-care:ti,ab OR domiciliary-care:ti,ab OR home-health-care:ti,ab OR formal-care:ti,ab OR home-nursing:ti,ab OR nursing-care:ti,ab OR home-health-nursing:ti,ab OR health services for the aged:ti,ab OR 'elderly care'/exp OR 'Home Care'/exp OR 'home health agency'/exp) AND (medication-management:ti,ab OR medication-reconciliation:ti,ab OR medical-analysis:ti,ab OR pharmaceutical-intervention:ti,ab OR pharmaceutical-service:ti,ab OR medication-regimen:ti,ab OR therapy-management:ti,ab OR medication-review:ti,ab OR pharmacist-recommendations:ti,ab OR drug-utilization-review:ti,ab OR drug-management:ti,ab OR medication-safety:ti,ab OR drug-analysis:ti,ab OR prescription-plan:ti,ab OR medication-plan:ti,ab OR drug-review:ti,ab OR brown-bag:ti,ab OR ‘medication therapy management‘/exp OR ‘risk assessment‘/exp OR ‘drug utilization review ‘/exp OR ‘medication review’/exp OR ‘pharmacist intervention’/exp) AND (pharmacist:ti,ab OR Pharmac*:ti,ab OR Interdisciplinary-health-teams:ti,ab OR 'pharmacist'/exp)

Figure S2.1: PRISMA 2020 [1] flow diagram.

Abbreviations: n, number

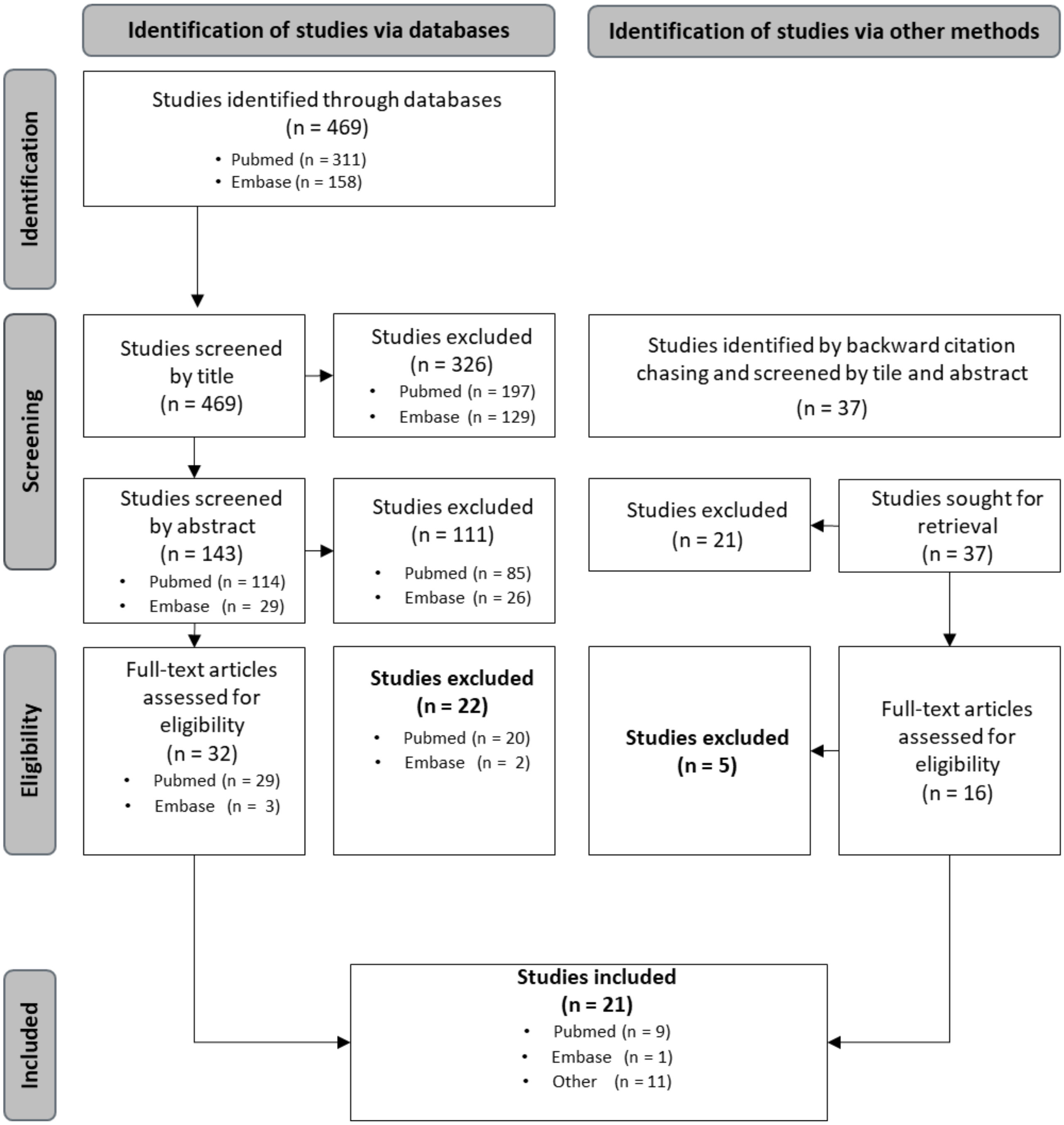

1. Tricco AC, Lillie E, Zarin W, O'Brien KK, Colquhoun H, Levac D, Moher D, Peters MDJ, Horsley T, Weeks L, et al. PRISMA Extension for Scoping Reviews (PRISMA-ScR): Checklist and Explanation. Ann Intern Med. 2018;169(7):467–73

Supplementary Material S4

Overview of 25 included tools assessing the risk of elderly patients for medication-related problems or prioritize them for clinical pharmacy services

- References marked blue:
  Tools to identify patients at increased risk for a medication-related problem or to prioritize patients for receipt of clinical pharmacy services
- References marked yellow: Lists from Europe with PIMs for geriatric patients.

	Reference	Name of the tool	Target audience of the tool	Aim of the tool	Development of the tool	Included criteria, cut off	Validation	Correlation
**1**	Parameswaran Nair(1) 2016 Australia	PADR-EC Score<br><br>Prediction of Hospitalization due to Adverse Drug Reactions in Elderly Community-Dwelling Patients score	Community-dwelling patients<br><br>≥65 years	Prediction of ADR-related hospitalization	hospital admission due to definite or probable ADR<br><br>March 2014 - March 2015<br><br>115 patients (mean age: 80.1 ± 7.7 years)<br><br>Identification of variables being associated with an ADR by univariate analysis (Fisher's exact test) and binary logistic regression model	Drug changes in the preceding 3 months (2 points)<br>Renal failure, GFR <60ml/min (2 points)<br>Dementia (2 points)<br>Number of antihypertensives<br>1-2 (3 points)<br>≥3 (5 points)<br>Anticholinergics (2 points)<br><br>Cut off ≥ 6 points (almost 3 times the risk of ADR-related hospitalization compared to those scoring <6)	Internal validation	Sensitivity of the score 72%<br>Specificity of the score 58%<br>ROC 0.67 (95% CI 0.56-0.78)
**2**	Nyborg(2) 2015 Norway	NORGEP-NH<br><br>Norwegian General Practice – Nursing Home criteria	Nursing home residents<br><br>>70 years	explicit criteria for pharmacologically inappropriate medication use to serve as a tool in the prescribing process and in medication reviews	27 proposed criteria based on Norwegian General Practice (NORGEP) criteria, literature, clinical experience<br><br>Three-round web-based Delphi consensus process (49 panellists (nursing home physicians, members of the Clinical Reference Group for Nursing Homes, geriatricians, clinical pharmacologists and pharmacists) 10-point Likert scale (clinical relevance))<br><br>August 2011 – March 2012<br><br>+7 criteria based on the panellists' suggestion in the first round	34 explicit criteria (drugs, dosages and drug combinations to be avoided)<br>11 single substance criteria (regular use should be avoided)<br>15 combination criteria (combinations to avoid)<br>8 deprescribing criteria (need for continued use should be reassessed)	N/A	

ADE=Adverse drug event, ADR=adverse drug reaction, DRP=Drug-related problems, EMR= electronic medical record, GFR=glomerular filtration rate, HbA1c=glycated haemoglobin, INR=international normalized ration; PIM=Potentially inappropriate medication

	Reference	Name of the tool	Target audience of the tool	Aim of the tool	Development of the tool	Included criteria, cut off	Validation	Correlation
**3**	Pammett(3)<br>2015<br>Canada	MRAQ (Medication Risk Assessment Questionnaire)<br><br>Self-administered screening questionnaire	Community pharmacy clients<br><br>≥18 years	Identification of patients at risk for drug therapy problems	Modification of a self-administered medication-risk questionnaire for use in elderly patients (10-item self-administered questionnaire by Levy Barenholtz [101]: items selected for inclusion in the questionnaire were based on published material and unpublished screening tools obtained through colleagues of the research team)<br><br>Inclusion of the 5 questions that correlated with drug related risk severity score and addition of some clarifications<br><br>**Evaluation**<br>Completion of the MRAQ by patient and a medication assessment by a pharmacist and determination of interrater agreement (Cohen's κ coefficient)<br><br>Comparison of the number of DTPs per patient categorized as high risk versus low risk by the MRAQ by using a Mann–Whitney U test and a Pearson Chi-square test	5-items (yes or no questions)<br><br>**1. Do you take 5 or more different medications on a regular basis? (including prescription and nonprescription products, vitamins, and herbals)**<br>2. Do you take 12 or more doses of medication each day?<br>**3. Are you currently taking medications for 3 or more medical conditions?**<br>4. Have your medications or the instructions on how to take them changed 4 or more times in the past year?<br>**5. Do you take any of the following medications? Carbamazepine, phenytoin, warfarin, rivaroxaban, dabigatran, apixaban, methotrexate, lithium, digoxin, any drug for chronic pain, insulin (any type), drugs to lower blood sugar**<br><br>**→high levels of interrater agreement**<br><br>High risk for DTPs if pharmacist researcher responded "yes" to three or more of the MRAQ questions	Evaluation of the screening questionnaire	High-risk patients identified by the MRAQ had significantly more DTPs

	Reference	Name of the tool	Target audience of the tool	Aim of the tool	Development of the tool	Included criteria, cut off	Validation	Correlation
**4**	Dimitrow(4)<br>2014<br>Finland	nurse-administered Drug-Related Problem Risk Assessment Tool (DRP-RAT)	Home Care<br><br>Home-dwelling clients<br><br>≥65 years	Identification of patients at high risk for ADRs or other DRPs and assisting in finding solutions to these problems	91 proposed criteria based on two systematic literature reviews and clinical expertise of the research group and a geriatrician not involved in the research group<br><br>Pilot Delphi round (2 authors, minor modifications)<br><br>Three-round Delphi consensus process (18 panelists (physicians, pharmacists, nurses))<br><br>September 2010 – December 2010<br><br>Consensus was reached on 48 items.<br><br>Retention of 11 items according to the research group's judgment (too important for the intended purpose to be excluded)	<u>59 items, divided into 4 sections</u><br>*1) basic client data* (Name, identity number, age, gender, does the client live alone?, **does the client have an up-to-date medication card/list?** who administers the client's medicines?<br><br>*2) potential risks for DRPs in medication use (38 items)*<br>Symptoms potentially suggestive of ADRs (yes/no, new (within last 4 weeks))<br>medicines potentially harmful or problematic for clients aged ≥65 years (medicines having a narrow therapeutic index, medicines for which regular monitoring would be necessary, medicines that are otherwise problematic for the aged)<br>items indicating risks posed by polypharmacy<br>initiation of a new medication<br>concomitant use of nonprescription medicines or vitamins with prescription medicines<br><br>*3) characteristics of the client's care and adherence (12 items)*<br>Information about the client's health<br>**Adherence to their therapeutic regimen**<br>involvement in one's care, the health care setting<br>health care units recently visited by the client<br>the number of care-taking physicians<br><br>*4) recommendations for actions to resolve DRPs*<br>7 interventions that practical nurses can recommend for resolving potential DRPs for those at risk based on the risk assessment resulting from the information gathered by the tool<br><br>**The two most important items for predicting DRP risks in the aged ≥65 years**	Content validation by Delphi process, feasibility evaluation among practical nurses in home care[102] and testing the validity of the tool in clinical practice[89]	In most cases PNs were able to find those patients at high risk for clinically significant DRPs by using the tool

	Reference	Name of the tool	Target audience of the tool	Aim of the tool	Development of the tool	Included criteria, cut off	Validation	Correlation
**5**	Makowsky(5) 2017 Canada	5-item EMR-MRAQ<br><br>EMR: electronic medical record<br>MRAQ: Medication risk assessment questionnaire	Primary care clinic<br><br>>18 years<br>≥2 medications	Electronic medical record screening for indicators of medication risk to identify patients in need of clinical pharmacist care	Adaption of a self-administered medication-risk questionnaire for use in elderly patients (10-item self-administered questionnaire by Levy Barenholtz [101]: items selected for inclusion in the questionnaire were based on published material and unpublished screening tools obtained through colleagues of the research team) for primary care ambulatory population in a previous study<br>10-item medication risk assessment questionnaire<br><br>Review of previous literature and evaluation of each of the 10-item self-administered medication risk assessment questionnaire criteria for ease of abstraction only 5 items were included, resulting in the creation of the 5-item EMR-MRAQ<br><br>**Internal Validation:**<br>Determination of correlation between risk assessment and health behavior/status scales<br><br>Calculation of area under the receiver operating characteristic curve (AUROC) to determine how well the score predicted health care utilization<br><br>Calculation of sensitivity, specificity, and positive and negative predictive values for all-cause emergency room visits/hospitalization and high number of clinic utilization and comparison to a 5 and 10 item self-assessment MRAQ<br><br>143 adults (median age 61 years)	1. ≥3 medical conditions<br>2. at least 1 target condition: anxiety, arthritis, asthma, coronary heart disease, chronic obstructive pulmonary disease, chronic pain, depression, diabetes mellitus, high blood pressure<br>3. ≥5 medications (including, prescription, non-prescription, herbal therapies)<br>4. ≥11 oral doses per day<br>5. ≥5 medication or instruction changes in the past year<br><br>patient at risk if ≥3 "yes" responses	Exploration of the ability to predict indicators of health behavior, health status, and health care utilization.	most effective in predicting all-cause emergency room visits/hospitalization (c-statistic = 0.69; 95% CI=0.57-0.81, sensitivity: 82.6%, specificity: 33.3%) and high clinic utilization (c-statistic = 0.77; 95% CI = 0.69-0.85, sensitivity 88.9%, specificity: 42.7%)

	Reference	Name of the tool	Target audience of the tool	Aim of the tool	Development of the tool	Included criteria, cut off	Validation	Correlation
**6**	Renom-Guiteras(6)<br>2015<br>Germany	EU(7)-PIM list<br><br>A list of potentially inappropriate medications consented by experts from seven European countries (Finland, Sweden, France, Spain, Germany, Netherlands, Estonia)	Older people living in European countries	Identification and international comparison of potentially inappropriate medication (PIM) prescribing patterns for older people and use as a guide in the clinical practice	preliminary PIM list based on German PRISCUS list of PIM, other PIM lists from the USA, Canada and France and a comprehensive literature search (30 experts on geriatric prescribing): 184 drugs<br><br>Structured expansion of the list by 8 experts (May 2012): 75 additional drugs<br><br>Two-round Delphi consensus process (round 1 26 and round 2 24 panellists, 5-point Likert scale (appropriateness))<br><br>October 2012 – May 2013<br><br>56 additional drugs (suggested by experts, updated Beers list), 3 non-PIM-drugs, 31 questionable PIM-drugs<br><br>Completion of a brief final survey to decide upon issues requiring further consensus by 12 experts: 282 drugs	275 chemical substances and 7 drug classes belonging to 55 therapeutic classes and 34 therapeutic groups<br>Some of the PIM concepts are dose-related or defined by length of use or drug regimen<br><br>The PIM list contains suggestions for dose adjustments and therapeutic alternatives<br><br>72 different PIMs were most frequently reported	N/A	
**7**	Alassaad(7)<br>2015<br>Sweden	80+ score	Hospital (acute internal medicine ward)<br><br>≥80 years	Prediction of risk of rehospitali-zation and mortality in the hospitalized elderly incorporating aspects of pharmaco-therapy	Time to rehospitalization or death during the year after discharge from hospital was measured.<br><br>368 patients (mean age 86.7 ± 4.1 years)<br><br>Selection of candidate variables among a large number of clinical and drug-specific variables (principal component analysis, stepwise Cox regression likelihood-ratio elimination procedure ($p<0.01$))<br><br>Construction of a point score system for risk estimation<br><br>Discriminatory ability of the score was assessed using C-statistic and compared with STOPP, START and MAI: 0.71 (80+), 0.57 (STOPP), 0.54 (START), 0.63 (MAI)	**4 clinical risk factors**<br>-impaired renal function: GFR 60-89ml/min (1 point)<br>GFR 30-59ml/min (2 points)<br>GFR <30ml/min (3 points)<br>-pulmonary disease (2 points)<br>-malignant disease (past or present) (1 point)<br>-living in a nursing home (1 point)<br><br>**3 drug specific risk factors**<br>-being prescribed an opioid (2 points)<br>-being prescribed a drug for peptic ulcer or gastroesophageal reflux disease (1 point)<br><br>-being prescribed an antidepressant drug (TCA excluded) →lower risk (-2 points)<br><br><u>Estimation of risk for the point total of the score</u><br>-2 points (0.1594), - 1 point (0.2207), 0 point (0.3010), 1 point (0.4021), 2 points (0.5223), 3 points (0.6539), 4 points (0.7821), 5 points (0.8879), 6 points (0.9568), 7 points (0.9890), 8 points (0.9985), 9 points (0.9999), 10 points (> 0.9999)	Internal validation	compared to STOPP, START and MAI →better discriminated risk than available tools for inappropriate prescribing

	Reference	Name of the tool	Target audience of the tool	Aim of the tool	Development of the tool	Included criteria, cut off	Validation	Correlation
**8**	Snyder(8)<br>2015<br>USA	Self-administered scale as a screening tool for MRPs	General, ambulatory patient population<br><br>≥18 years, taking at least 1 regularly scheduled prescription medication	Prediction of medication-related problems (MRPs) which may supplement other available patient data in developing patient eligibility criteria for medication therapy management	**Phase 1**: use of the 78-item pool of the Drug Therapy Concerns Questionnaire (DTC) to identify brief, self-administered scale for further testing<br><br>Completion of the 78 items by 394 patients (mean age 50.9 years) over a period of 4 months in 2009<br><br>Selection of items that maximized clinical utility for further investigation as a brief, self-administered scale, estimation of reliability and construction of validity of the resulting instrument<br><br>**Validation**<br>**Phase 2**: estimation of the utility of the brief scale for use as MRP predictor<br><br>Completion of the 9-item self-administered scale by 200 patients (mean age 64.8 years) between December 2011 - March 2013<br><br>Estimation of the criterion-related validity of the scale by counting the number of pharmacist-identified MRPs by medication review<br><br>Construction of a multivariate model to examine the influence of scale scores on MRPs after controlling for other significant variables (total number of vitamins, herbals, supplements; household income (comfortable, just enough to make ends meet, not enough to make ends meet); total daily medication doses)	9-items (5-point Likert scale)<br>1. Sometimes my medication has effects I do not like.<br>2. My medication does not seem to help that much.<br>**3. Sometimes I think I take too many medications.**<br>**4. I worry about drug interactions between the medications I take.**<br>5. I have trouble taking my medication the way I am supposed to.<br>6. Sometimes I feel worse after I take my medication.<br>7. Sometimes I think I may not be taking the right medication for my condition.<br>8. My medication interferes with my routine daily activities.<br>9. My medication is helping improve my condition.<br><br>**Best performing items**<br><br>score threshold of 15 points (items reverse scored as required) has a sensitivity of 81-86% and a specificity of 61% of identifying patients with at least one MRP	Internal validation	Higher scores were positively correlated with more pharmacist-identified MRPs

	Reference	Name of the tool	Target audience of the tool	Aim of the tool	Development of the tool	Included criteria, cut off	Validation	Correlation
**9**	Ploenzke(9) 2016 USA	CCTS<br><br>Composite Care Transitions Score	Veterans Health Administration (VHA) primary care population at transition of care<br><br>Age not specified	Identification of high-risk patients at transition of care for medication misadventures and those who might benefit from a pharmacist encounter	Identification and organization of patient-specific factors incurring high risk for medication errors based on literature review (English-language, U.S. health care-based literature)<br><br>Combination with a national stratification tool unique to the Veterans Health Administration (VHA) primary care population, the Care Assessment Need (CAN) score<br><br>Pooling and organization of the high-risk measures and forming a consensus by the project investigators on which criteria were to be included for risk assessment<br><br>Assignment of points to each risk factor based on clinical significance to pharmacy by the project investigators<br><br>Pilot study inclusion criteria<br>-discharge from the institution's hospital or transitional care unit in the past 7 days<br>-CAN score ≥95th percentile (represents a population of patients with an approximately 50% risk for hospitalization or death within the next year)<br><br>Exclusion criteria<br>-inpatient status in an assisted living housing, transitional care unit, hospice, or nursing home<br>-deemed inappropriate for a MTM determined by the investigators<br>-CCTS ≥10 (more likely to benefit from a physician mediated follow-up because of the complexity associated with end-of-life management and specialty care)	16 risk factors<br>age >65 years (1 point)<br>>10 active medications (2 points)<br>Readmissions within past 30d (1 point)<br>3 or more readmissions in past year (2 points)<br>last hospital stay >48h (2 points)<br>active anticoagulant prescription (warfarin, clopidogrel plus aspirin, dabigatran, rivaroxaban, apixaban) (1 point)<br>active digoxin prescription (1 point)<br>active hypoglycemic prescription (Insulin, glipizide, glyburide, glimepiride) (1 point)<br>active opioid prescription (morphine, fentanyl, hydrocodone in combination, oxycodone alone or in combination) (1 point)<br>depression diagnosis (1 point)<br>pneumonia diagnosis (1 point)<br>Parkinson disease (1 point)<br>Multiple sclerosis (1 point)<br>Other dementia diagnosis (traumatic brain injury, vascular) (1 point)<br>Heart failure diagnosis (1 point)<br>Myocardial infarction diagnosis (1 point)	Evaluation	Stratifying patient by means of the Composite Care Transition Score correctly identified high-risks patients with multiple medication concerns

	Reference	Name of the tool	Target audience of the tool	Aim of the tool	Development of the tool	Included criteria, cut off	Validation	Correlation
**10**	Rovers(10)<br>2012<br>USA	Drug therapy self-assessment tool	Senior center<br><br>Elderly patients	Identification of patients at risk for drug therapy problems (DTPs) and motivate those at risk to participate in a personal pharmaco-therapy consultation with a pharmacist	Conceptually and empirically linkage of the self-assessment items to one or more DTPs based on the literature<br><br>Empirically assignment of cut-off scores for possible DTPs (making the assignments empirically was decided to be acceptable since any DTPs experienced by the patient would be formally identified during the personal consultation and the tool was designed as a device to motivate participants to schedule a consultation)	12-items (5-point scale 1 (never) to 5 (always))<br><br>*Group 1 count (count number of answers where you selected either a 1 or 2 response)*<br>**Inappropriate adherence**<br>I know why I am taking each of my medications.<br>I take my medications the way I am supposed to.<br>I know how I am supposed to take all my medications.<br>**Unnecessary drug therapy**<br>I think I need the drugs my doctor prescribes.<br>**Wrong drug**<br>I think my medications work well for me.<br><br>*Group 2 count (count number of answers where you selected either a 4 or 5 response)*<br>**Adverse drug reaction**<br>I get side effects from medications.<br>I am concerned about drug interactions and side effects.<br>**Dosage too high or too low**<br>My doctor has to adjust the dose of my medications up or down.<br>**Wrong drug**<br>My doctor changes me from one medication to a new medication of the same illness.<br>**Inappropriate adherence**<br>I only get part of my prescription filled because I cannot afford the full amount.<br>**Needs additional drug therapy**<br>I use over-the counter medications even though I believe I should have seen a doctor for a prescription.<br>I have symptoms or complaints that I have not seen my doctor about.<br><br>Score of 0: suggesting no DTPs<br>Score of 1 to 3: suggesting possible concerns or questions about drug therapy<br>Score of ≥4: suggesting the participant had one or more DTPs	Evaluation of the tool, but reliability and validity of the self-assessment tool to actually identify DTPs was of secondary interest!	For participants whose DTPs were later identified during their personal consultation, the score on the self-assessment tool was found to be positively and significantly correlated with the number of DTPs eventually found

	Reference	Name of the tool	Target audience of the tool	Aim of the tool	Development of the tool	Included criteria, cut off	Validation	Correlation
**11**	Gusdal(11)<br>2011<br>Sweden	SMA tool<br><br>Safe Medication Assessment tool	Primary health care, Home Care<br><br>Nurse-administered<br><br>Elderly patients	Identification of factors highly related to unsafe medication management	Development of the SMA pilot based on findings and recommendations from the workgroup for better use of medicines reports that highlight problems with adherence concordance, and medication management and suggests measurements, which lead to appropriate care and better outcomes<br><br>Check, revision and approval of the SMA pilot<br><br>Rating of each observation's value of relevance and content using a three-point scale (high, acceptable, low)<br><br>Retention of those observations which the district nurses considered to have a high or acceptable value of relevance and content	Screening<br>The patient:<br>1. Can describe all medication used<br>2. Can describe each illness for which each medication is prescribed<br>3. Has assistance in dispensing medication dosage<br>4. Uses less than three drugs<br>→1 no →continue<br><br>Observations (yes or no)<br>The patient:<br>5. Can describe each medicine's administration form, potency, and dosage<br>6. Has prescribers of medication from more than two medical units<br>7. Has (in your opinion) a suspected risk of interactions between drugs<br>8. Independently takes medicine out of a bottle or dose dispenser<br>9. Has a complex medication regimen<br>10. Can swallow medicine<br>11. Has a method for remembering to take his or her medicine<br>12. Has a storage method for his or her medicine<br>13. Has symptoms that can be presumed to be adverse effects of medication<br>14. Intentionally alters prescribed dosages without consulting prescribers<br>15. Places trust in the prescriber<br>16. Experiences having an excess of medication<br><br>Life style; the patient:<br>Has (in your opinion) alcohol-related problems<br>Lives alone	Exploration of the capability of the SMA tool in identifying factors highly related to unsafe medication management	SMA had the capability to identify factors highly related to unsafe medication management

	Reference	Name of the tool	Target audience of the tool	Aim of the tool	Development of the tool	Included criteria, cut off	Validation	Correlation
**12**	Singh(12)<br>2009<br>USA	Trigger tool for identifying ADE among older adults	Ambulatory primary care practices<br><br>Older adults	Identification of adverse drug events (ADEs) among older adults	Use of an existing trigger tool (Gurwitz, 58 triggers) and addition of triggers for “Emergency room visit”, “Unplanned hospitalization”, “Death” and “Abrupt medication stop”<br><br>Elimination of ICD-9-CM triggers because they were rarely found in written charts (23 items)	39 items, mentioned those, that are applicable in Home Care<br><br>*Serum drug levels, to high*<br>Carbamazepin, Ciclosporine, Digoxin, Phenytoin, Phenobarbital, Theophylline, Valproic acid<br>*Different laboratory values*<br>**Medications stopped**<br>Antidotes/treatments<br>Other life events (**emergency room visit**, **hospitalization** (unplanned))<br><br>**Triggers with the highest positive predictive values**	Evaluation and external validation[90]	External validation: Sensitivity of the trigger tool 11.2% Specificity of the trigger tool 99.7% PPV 85.0%; NPV 88.1%
**13**	O’Mahony(13)<br>2015<br>Ireland	STOPP/START criteria<br><br>Screening Tool of Older Person's Prescriptions and Screening Tool to Alert doctors to Right Treatment	Target group not specified<br><br>≥65 years	Screening of older persons' prescriptions incorporating criteria for potentially inappropriate drugs and criteria for potentially appropriate, indicated drugs	Review of the 2008 STOPP/START criteria (87 criteria): Addition of new evidence-based criteria, removal of any obsolete criteria and reassessment of the evidence base of the 2008 criteria by a thorough literature review<br><br>127 proposed criteria<br><br>Two-round Delphi validation round (19 panellists from 13 European countries with recognized expertise in Geriatric Medicine and pharmacotherapy in older people) 6-point Likert scale (0: don’t know, 1: strongly agree, 5: strongly disagree))<br><br>Consensus was not reached for 10 criteria	114 STOPP/START criteria<br><br>80 STOPP criteria<br>34 START criteria	N/A	

	Reference	Name of the tool	Target audience of the tool	Aim of the tool	Development of the tool	Included criteria, cut off	Validation	Correlation
**14**	Cullinan(14)<br>2016<br>Ireland	FI score<br><br>Frailty index score	Hospital<br><br>Older patients	Identification of potentially inappropriate prescribing (PIP) and adverse drug reaction risks in older patients	Use of a database compiled in a previous study:<br>737 patients underwent a comprehensive geriatric assessment on admission to hospital (physical and mental wellbeing)<br><br>Assessment of suitability of the variables from the database for inclusion in the frailty index and construction of the score<br><br>Correlation of the FI score/number of medications with the number of breaches of STOPP criteria (version 1)<br><br>Quantification of an instance of PIP regarding dependence on frailty/number of medications quantified by chi-square test.<br><br>Calculation of the risk associated with being in either group by odds ratio (OR)	34 variables (yes (1) or no (0) statements)<br>1. needs help grooming 2. needs help using toilet<br>3. needs help feeding 4. needs help with transfer<br>5. needs help mobilizing 6. needs help dressing<br>7. needs help with stairs 8. needs help bathing<br>9. dementia/cognitive impairment 10. on-going constipation<br>11. fall in the last 3 months 12. difficulty swallowing<br>13. trouble sleeping 14. previous myocardial infarction<br>15. hypertension 16. congestive cardiac failure<br>17. peripheral vascular disease 18. cerebrovascular disease<br>19. chronic pulmonary disease 20. rheumatological disease<br>21. peptic ulcer disease 22. mild or moderate liver disease<br>23. diabetes 24. diabetes with complications<br>25. hemiplegia or paraplegia 26. renal disease<br>26. any malignancy 28. moderate or severe liver disease<br>29. metastatic solid tumour 30. inconctinence (bowels)<br>31. inconctinence (bladder) 32. self-reported depression<br>33. ≥6 medications 34. abbreviate mental test score<br><br>FI score is calculated by the sum of yes-statement divided by 34. FI score at which patients had at least one instance of PIP on their prescription: 0.16<br><br>Above this threshold patients were twice as likely to experience PIP (OR = 2.6, P < 0.0001) and twice as likely to develop an ADR (OR = 2.1, P < 0.0001)<br><br>Patients taking more than six medications were 3 times more likely to experience PIP	Determination of the association between a patient's FI score, the number of instances of PIP on the prescription and the likelihood of developing an ADR	Significant correlation between FI score instances of PIP (R = 0.92)

	Reference	Name of the tool	Target audience of the tool	Aim of the tool	Development of the tool	Included criteria, cut off	Validation	Correlation
**15**	Kaufmann(15) 2018 Switzerland	DART<br><br>Drug-Associated Risk Tool	Hospital<br><br>≥18 years	Identification of patients at risk for drug-related problems (DRPs) for targeted pharmaceu-tical care during the hospital stay and on discharge	Creation of a self-assessment questionnaire out of 27 risk factors identified through literature review, Nominal Group Technique with practitioners and a Delphi questionnaire in a previous study[66]<br><br>Transformation of risk factors into questions and statements<br><br>Validation of DART<br>Evaluation of sensitivity, specificity and prevalence of each question of DART by comparison of the subjective patients' answers in the DART with objective data from medical records, Beliefs about Medicines Questionnaire and Micro-Mental Test<br><br>2 mid-size hospitals, 164 patients (median age 74 years)<br><br>Revision of statements with low sensitivity and possible poor patient understanding (heart insufficiency, renal impairment, liver impairment)<br><br>Revalidation of DART<br>Evaluation of sensitivity, specificity and prevalence of the new statements of DART by comparison of the subjective patients' answers in the DART with objective data from medical records<br><br>2 mid-size hospitals, 31 patients (median age 82 years)	Items concerning the presence of diseases and high-risk medicines, reflecting the patient's attitude towards his/her medicines and statements about medication management and handling difficulties<br><br>Included risk factors:<br>Language issues (e.g. migration background)<br><br>Renal impairment (GFR <60ml/min), Hepatic impairment, Chronic cardiac disease, Chronic respiratory disease, Diabetes, Cognitive impairment/dementia<br><br>Patient takes medications besides the prescribed ones (e.g. over the counter, vitamin supplementation), Polypharmacy (number of drugs >5), Antiepileptic, anticoagulants, NSAIDs, combination of NSAIDs and anticoagulants, digoxin, corticosteroids, diuretics, tricyclic antidepressants, anticholinergic drugs, benzodiazepines, opiates/opioids, oral antidiabetics/insulin, medication with narrow therapeutic range<br><br>Non-adherence, Earlier experience of adverse drug reactions, Missing information, partial knowledge of the patient, the patient, does not understand the goal of the therapy<br><br>Impaired manual skills-causing handling difficulties, Visual impairment/impaired eyesight, Medication that is difficult to handle	validation of the tool regarding feasibility, acceptability and reliability of the patients' answers	specificity of DART: 88%, sensitivity of DART: 67%; specificity of the statements: mostly high sensitivity of the statements: higher in statements concerning diseases that require regular disease control and attention to self-care and drug management

	Reference	Name of the tool	Target audience of the tool	Aim of the tool	Development of the tool	Included criteria, cut off	Validation	Correlation
**16**	Onder(16)<br>2010<br>Italy	GerontoNet ADR Risk Score<br><br>GerontoNet Adverse Drug Reaction Risk Score	hospital<br><br>Elderly patients	Identification of elderly patients who are at increased risk for an ADR	Hospital admission of patients aged ≥65 years (data from Italian Group of Pharmacoepidemiology in the Elderly (GIFA database))<br><br>May – June and September – October in 1993, 1995 and 1997<br><br>5936 patients (mean age: 78.0 ± 7.2 years)<br><br>Identification of 383 patients experiencing an ADR during hospital stay defined as probable or definite (due to information of the nurses and the attending physician and medical and nursing records)<br><br>Identification of variables associated with ADRs by a combination of univariate analysis (X^2 test, $p \leq 0.10$) and multivariate logistic regression analysis ($p \leq 0.10$)	Risk factors with scored points<br>≥ 8 drugs (4 points); 5-7 drugs (1 point)<br>previous ADR (2 points)<br>heart failure (1 point)<br>liver disease (1 point)<br>≥4 comorbid conditions (1 point)<br>renal failure: GFR < 60ml/min (1 point)<br><br>Youden's Index was found to be greatest when the cut-off score was between 3 and 4	Internal and external validation [91]	Internal validation: sensitivity: 68%, specificity: 65%, AUROC: 0.73 (95% CI 0.69 - 0.73)<br>External validation: 1. Type A ADRs (dose dependent and predictable): AUC: 0.69 (95% CI 0.60–0.77)<br>2. probably or definitely related to drug use: AUC: 0.64 (95% CI 0.55–0.74)

	Reference	Name of the tool	Target audience of the tool	Aim of the tool	Development of the tool	Included criteria, cut off	Validation	Correlation
**17**	Falconer(17)<br>2014<br>New Zealand	ART<br><br>Assessment of Risk Tool	Hospital<br><br>Age not specified	Electronic prioritization of patients for pharmacist interventions by ADE risk assessment	Using of specific clinical criteria (flags) known to be associated with increased risks of ADEs and MEs due to international literature to create the tool<br><br>Review of the flags by senior pharmacists at Middlemore Hospital and derivation of an appropriate score for each flag through a group consensus process<br><br>Identification of appropriate electronic data sources available within the hospital to extract the necessary information	38 clinical flags assigned with a weighted score from 1 to 10 (in brackets behind the flag) resulting in a patient's total risk score mentioned those, that are applicable in Home Care.<br><br>**Patient profile**<br>Age >75 years (10), no registered general practitioner (5), poor medication compliance (1), English difficulty (1)<br><br>**Patient encounter**<br>Patients with >4 emergency care/inpatient admissions in previous 12 months (8), >2 outpatient visits to different specialties in previous 6 months (8), mental health history within previous 12 months (8), patient under care of hematology or renal service (6), readmission to hospital in <7 days (4) or <30 days (2)<br><br>**Clinical profile – patients with chronic disease**<br>Previously (within past 12 months) enrolled in chronic care management in diabetes (4), chronic obstructive pulmonary disease (4), congestive heart failure (4), cerebrovascular disease (4)<br><br>**High-risk medications**<br>>8 regular medications (10), antiepileptic medication (2), anticoagulant medication (2), >3 cardiovascular medications (2), antidiabetic medication (2), >1 opioid medication (2), medication requiring therapeutic drug monitoring (2), patients aged >80 years and admission source for current encounter designated in potentially inappropriate medications, a rest home or private hospital or specified other hospitals and taking >8 regular medications and received specified high-risk medications in past 30 days (10)<br><br>**Laboratory values**<br>Poor renal function (e.g. GFR <30ml/min) (8), coagulation risk (e.g. INR >3.5/ aPTT >100s) (4), HbA1c >64mmol/mol in last 90 days (4)<br><br>Categorization of the patients<br>Scores >22: high risk for MEs and ADEs<br>Scores 11-22: medium risk for MEs and ADEs<br>Scores ≤10: low risk for MEs and ADEs	external validation [92]	Patients in the high-risk group had a significantly greater number of unintentional medication discrepancies than those in the medium- and low-risk groups. significant association with risk for « more than eight admission medications» (OR=3.7) and « readmission within 30 days » (OR=6.8), p<0.0001

	Reference	Name of the tool	Target audience of the tool	Aim of the tool	Development of the tool	Included criteria, cut off	Validation	Correlation
**18**	Doucette(18)<br>2013<br>USA	MUSE tool<br><br>Medication User Self-Evaluation Tool	Setting and age not specified<br><br>Medicare Part D beneficiaries	Identification of Medicare beneficiaries likely to benefit from medication therapy management (MTM) services	Creation of 225 patient medication profiles of a random sample from a survey of Medicare beneficiaries (set of 10 questions including demographic, characteristics, responses to adherence questions, and reported symptoms developed based on the literature)<br><br>Judgment of the likelihood that the patients would benefit from an MTM visit in the next 3 months by 3 clinical pharmacists (low, moderate, or high)<br><br>Use of 150 patient profiles (mean age 72.44 ± 6.00 years) for model calibration<br><br>Fit of ordinal logistic regression models to predict the likelihood of benefit from an MTM visit by using different combinations of potential MUSE items<br><br>Selection of final model based on the Akaike information criterion and the percent agreement between model prediction and expert judgments in the validation data	7-items<br>1. How many prescription medications do you take regularly? (number)<br>2. During the past month, have you forgotten to take your medication(s) for any reason? (Yes/no)<br>3. In the past year, have you not filled a new prescription or stopped taking a prescription medication because of the cost? (Yes/no)<br>4. In a typical month, from how many pharmacies do you get prescriptions, including mail order? (number)<br>5. Have you been admitted into a hospital in the past 6 months? (Yes/no)<br>6. How many physicians have prescribed medications for you in the past year? (number)<br>7. Please tell me the number of medical conditions for which you are receiving treatment. (number)	Internal validation	Prediction of benefit from an MTM for 7 of 10 cases
**19**	Tangii-suran(19)<br>2014<br>UK	BADRI model<br><br>Brighton Adverse Drug Reactions Risk Model	Hospital<br><br>generalizable across populations and appropriate for predicting ADR risk in the oldest old (≥85 years)	Identification of patients at risk of developing an ADR to enable healthcare staff to put measures in place to reduce the risk of such an event developing	Hospital admission to one of 4 wards (elderly care and stroke) of patients aged ≥65 years<br><br>two 3-month periods:<br>January – March in 2007 and 2008; September 2008 – February 2009<br><br>690 patients (mean age: 84.3 years (range 65-103 years))<br><br>Confirmation of 95 reports of ADR by independent review in these patients<br><br>Identification of clinical risk factors for developing an ADR by a combination of univariate analysis and multivariate binary logistic regression analysis	Risk factors with scored points<br>≥8 drugs (1 point)<br>Hyperlipidemia (1 point)<br>high white blood cell count on admission (1 point)<br>use of anti-diabetic agents (1 point)<br>length of stay ≥12 days (1 point)<br><br>cut-off score >1	Internal validation and external validation	Internal validation: sensitivity: 80%, specificity: 55%, AUROC: 0.74 (95% CI 0.68 - 0.79); external validation: sensitivity: 84%, specificity: 43%, AUROC: 0.73 (95% CI 0.66 - 0.80)

	Reference	Name of the tool	Target audience of the tool	Aim of the tool	Development of the tool	Included criteria, cut off	Validation	Correlation
**20**	Akbarov(20)<br>2015<br>UK	Indicators of unsafe medication practice	Primary care<br><br>>18 years	Surveillance of primary care medication safety with integrated primary and secondary care electronic health records	Consideration of 18 prescribing and 4 monitoring indicators most likely associated with preventable drug-related morbidity mentioned in literature and existing tools.<br><br>Conduction of a cross-sectional study using linked records of patients served by one hospital and over 50 general practices in UK<br><br>205519 patients (mean age: 38.89 ± 7.96 years)<br><br>Use of mixed-effects logistic models, relating prescribing safety indicators to potential determinants	<u>22 medication safety indicators</u><br>18 prescribing and 4 monitoring indicators	N/A	
**21**	Resar(21)<br>2016<br>USA	Outpatient adverse event trigger tool of Institute for Healthcare Improvement (IHI)	outpatients<br><br>>18 years	Representing opportunities that may lead to an adverse event to alert reviewers to focus further investigation in order to determine whether an adverse event actually occurred	Development of the triggers using data (claims data) to create a test set<br><br>Confirmation of the initial best guesses by testing over a wide range of outpatient interactions<br><br>Outpatient triggers are described as “life events”, which are contact points with health care where adverse events could either be observed or caused by the care received	<u>11 triggers</u><br>New diagnosis of cancer<br>Nursing home placement<br>Admission and discharge from the hospital<br>≥2 consultants in a year of review<br>Surgical procedure<br>Emergency department visit<br>>5 medications<br>Physician change<br>Complaint letter<br>≥3 nursing calls in one week<br>Abnormal lab value (e.g. INR >6, Glucose <50)<br><br><u>Possible additional triggers:</u><br>Abrupt medication stop<br>Sudden change in treatment or care plan<br>Outpatient code or arrest	Triggers have been tested	

	Reference	Name of the tool	Target audience of the tool	Aim of the tool	Development of the tool	Included criteria, cut off	Validation	Correlation
**22**	Barnett(22)<br>2011<br>UK	Prevent tool[,103]	Different settings<br><br>Age not specified	Identification of patients at risk of preventable medicines-related readmission with unmanaged complex pharmaceu-tical issues where the risk is modifiable through pharmaceu-tical care	Development of the tool using an iterative process<br><br>Published tools, including the Fleetwood model, the Combined Predictive Model and STOPP-START, evidence from literature, action research and expert opinion were used.<br><br>Refinement of the tool through reflective practice, root cause analysis and patient feedback.	Physical impairment<br>Difficulties with swallowing, impaired dexterity, poor vision, hard of hearing, poor mobility which will impact them taking medication<br><br>Frailty<br><br>Adherence/issues/compliance support<br>Bad compliance, patient has decided to stop taking the medicines<br><br>Cognitive impairment<br>Condition which affects their memory (e.g. delirium, dementia)<br><br>New diagnosis/exacerbation of disease<br>Admission related to poor management of medication of a long term clinical condition or deterioration of organ system function (renal, cardiac)<br>Previous admission within 30 days<br>Depression, high level of stress, other mental health, alcohol or drug abuse<br><br>Medicines related admission/risk from specific medicines which the patient is unable to manage<br>Anticoagulants/antiplatelets, insulin/oral hypoglycaemics, NSAID, benzodiazepine, antihypertensives, diuretics, beta blockers, opioids, methotrexate, injectable medicines, drugs requiring therapeutic drug monitoring esp. with no monitoring, steroids complex of medicine regimen, recent stop, start or change in medicines or polypharmacy<br><br>Cultural/social<br>cannot manage daily activities independently/has carers to help with daily activities but not medicines.<br>Patient has cultural beliefs around illness and treatment impacting medication adherence<br>Patient has social issues such as no fixed abode, unkempt etc which impacts them taking medication<br>Smoker	Not yet validated	

	Reference	Name of the tool	Target audience of the tool	Aim of the tool	Development of the tool	Included criteria, cut off	Validation	Correlation
**23**	NHS West Midlands(23)<br>2012<br>UK	Risk indicators for Medicines-related Problems	General practice, community pharmacy, hospital, care home<br><br>Age not specified	Identification of people who might be at an increased risk of medicines-related problems	Identification of risk factors following a search of published literature and review by a group of clinical professionals	Risk factors (score 1 for each risk factor present)<br>1. age >65 years<br>2. taking >5 medicines or >12 doses of medicines each day<br>3. recent change in medicines (medicine added, medicine stopped, dose changed)<br>4. higher risk medicines (NSAID, Aspirin, diuretic, ACE-inhibitor, angiotensin-II-receptor-antagonist, digoxin, warfarin, drugs for diabetes including insulin, lithium, methotrexate)<br>5. difficulty in taking medicines as prescribed eg. swallowing problems, forgetfulness, unable to open medicines containers etc.<br>6. kidney or liver problems<br>7. dependant on support to take medicines<br>8. has had medicines-related problems in the past<br><br>Scores 12-18: higher risk<br>Scores 7-12: moderate risk<br>Scores 0-6: lower risk		
**24**	Holt(24)<br>2010<br>Germany	Priscus list	Setting not specified<br><br>Elderly living in Germany	List of potentially inappropriate medications (PIMs) for the elderly for use in Germany	Creation of a preliminary PIM list suitable for the German Market based on a selective literature search and a qualitative analysis of published international PIM lists<br><br>Modified two-round Internet-based Delphi consensus process (25 panellists (represented 8 different specialties: geriatric medicine, clinical pharmacology, general practice, internal medicine, pain therapy, neurology, psychiatry, and pharmacy) 5-point Likert scale (rating appropriateness))<br><br>Recommendations of monitoring parameters, dose adjustments, alternative, predominantly pharmacological, treatment alternatives and any comorbidities that would elevate the risk of adverse events for each drug by the panellists<br><br>Start in December 2008<br><br>Development of the final German PIM list	List of 83 drugs (in a total of 18 drug classes) rated as PIMs<br><br>Including recommendations for clinical practice if the administration of a PIM is clinically necessary (e.g. monitoring of laboratory values and dose adaptation)<br><br>Possible therapeutic alternatives are mentioned	N/A	

	Reference	Name of the tool	Target audience of the tool	Aim of the tool	Development of the tool	Included criteria, cut off	Validation	Correlation
**25**	Tommelein(25) 2016 Belgium	GheOP^{3}S-tool<br><br>Ghent Older People's Prescriptions community Pharmacy Screening tool	Community pharmacy, European market<br><br>Older patients	Detection of relevant potentially inappropriate prescribing (PIP) in older patients' prescriptions by an explicit screening tool	Retrieval of 18 published lists of PIP mentioning different items by a literature review, including an alternative therapeutic option for each PIP item relying on existing evidence: selection of 121 items<br><br>Two-round RAND/UCLA (Research and Development/University of California, Los Angeles) process (11 panellists (clinical pharmacists, general practitioners, academics, community pharmacist and physician))<br><br>February 2013 – May 2013<br><br>a first written evaluation round: rating of the clinical value of a check of the items on a 9-point Likert scale (1: no added clinical value, 9: high added clinical value)<br><br>a second face-to-face evaluation round: focused discussion on newly suggested items and on items for which there was "disagreement" among the panellists: 99 items<br><br>selection of those item that are applicable in the contemporary community pharmacy (two-round Delphi consensus process (7 Belgian community pharmacists)): 83 items	83 items<br>Part 1: potentially inappropriate drugs, independent of diagnosis<br>Part 2: potentially inappropriate drugs, dependent on diagnosis<br>Part 3: potential prescribing omissions<br>Part 4: drug-drug interactions of specific relevance<br>Part 5: general care-related items to be addressed in the community pharmacy	N/A	

References

1. Parameswaran Nair N, Chalmers L, Connolly M, Bereznicki BJ, Peterson GM, Curtain C, et al. Prediction of Hospitalization due to Adverse Drug Reactions in Elderly Community-Dwelling Patients (The PADR-EC Score). PLoS One. 2016;11(10):e0165757.
2. Nyborg G, Straand J, Klovning A, Brekke M. The Norwegian General Practice--Nursing Home criteria (NORGEP-NH) for potentially inappropriate medication use: A web-based Delphi study. Scand J Prim Health Care. 2015;33(2):134-41.
3. Pammett RT, Blackburn D, Taylor J, Mansell K, Kwan D, Papoushek C, et al. Evaluation of a Community Pharmacy-Based Screening Questionnaire to Identify Patients at Risk for Drug Therapy Problems. Pharmacotherapy. 2015;35(9):881-6.
4. Dimitrow MS, Mykkanen SI, Leikola SN, Kivela SL, Lyles A, Airaksinen MS. Content validation of a tool for assessing risks for drug-related problems to be used by practical nurses caring for home-dwelling clients aged >/=65 years: a Delphi survey. Eur J Clin Pharmacol. 2014;70(8):991-1002.
5. Makowsky MJ, Cor K, Wong T. Exploring Electronic Medical Record and Self-Administered Medication Risk Screening Tools in a Primary Care Clinic. J Manag Care Spec Pharm. 2017;23(5):566-72.
6. Renom-Guiteras A, Meyer G, Thurmann PA. The EU(7)-PIM list: a list of potentially inappropriate medications for older people consented by experts from seven European countries. Eur J Clin Pharmacol. 2015;71(7):861-75.
7. Alassaad A, Melhus H, Hammarlund-Udenaes M, Bertilsson M, Gillespie U, Sundstrom J. A tool for prediction of risk of rehospitalisation and mortality in the hospitalised elderly: secondary analysis of clinical trial data. BMJ Open. 2015;5(2):e007259.
8. Snyder ME, Pater KS, Frail CK, Hudmon KS, Doebbeling BN, Smith RB. Utility of a brief screening tool for medication-related problems. Res Social Adm Pharm. 2015;11(2):253-64.
9. Ploenzke C, Kemp T, Naidl T, Marraffa R, Bolduc J. Design and implementation of a targeted approach for pharmacist-mediated medication management at care transitions. J Am Pharm Assoc (2003). 2016;56(3):303-9.
10. Rovers J, Hagel H. Self-assessment tool for screening patients at risk for drug therapy problems. J Am Pharm Assoc (2003). 2012;52(5):646-52.
11. Gusdal AK, Beckman C, Wahlstrom R, Tornkvist L. District nurses' use for an assessment tool in their daily work with elderly patients' medication management. Scand J Public Health. 2011;39(4):354-60.
12. Singh R, McLean-Plunckett EA, Kee R, Wisniewski A, Cadzow R, Okazaki S, et al. Experience with a trigger tool for identifying adverse drug events among older adults in ambulatory primary care. Qual Saf Health Care. 2009;18(3):199-204.
13. O'Mahony D, O'Sullivan D, Byrne S, O'Connor MN, Ryan C, Gallagher P. STOPP/START criteria for potentially inappropriate prescribing in older people: version 2. Age Ageing. 2015;44(2):213-8.
14. Cullinan S, O'Mahony D, O'Sullivan D, Byrne S. Use of a frailty index to identify potentially inappropriate prescribing and adverse drug reaction risks in older patients. Age Ageing. 2016;45(1):115-20.
15. Kaufmann CP, Stampfli D, Mory N, Hersberger KE, Lampert ML. Drug-Associated Risk Tool: development and validation of a self-assessment questionnaire to screen for hospitalised patients at risk for drug-related problems. BMJ Open. 2018;8(3):e016610.
16. Onder G, Petrovic M, Tangiisuran B, Meinardi MC, Markito-Notenboom WP, Somers A, et al. Development and validation of a score to assess risk of adverse drug reactions among in-hospital patients 65 years or older: the GerontoNet ADR risk score. Arch Intern Med. 2010;170(13):1142-8.
17. Falconer N, Nand S, Liow D, Jackson A, Seddon M. Development of an electronic patient prioritization tool for clinical pharmacist interventions. Am J Health Syst Pharm. 2014;71(4):311-20.
18. Doucette WR, Chang EH, Pendergast JF, Wright KB, Chrischilles EA, Farris KB. Development and initial assessment of the medication user self-evaluation (MUSE) tool. Clin Ther. 2013;35(3):344-50.
19. Tangiisuran B, Scutt G, Stevenson J, Wright J, Onder G, Petrovic M, et al. Development and validation of a risk model for predicting adverse drug reactions in older people during hospital stay: Brighton Adverse Drug Reactions Risk (BADRI) model. PLoS One. 2014;9(10):e111254.
20. Akbarov A, Kontopantelis E, Sperrin M, Stocks SJ, Williams R, Rodgers S, et al. Primary Care Medication Safety Surveillance with Integrated Primary and Secondary Care Electronic Health Records: A Cross-Sectional Study. Drug Saf. 2015;38(7):671-82.

21. Resar R. Outpatient Adverse Event Trigger Tool: Institute for Healthcare Improvement in association with Kaiser Permanente and Baylor Health Care System: Institute for Healthcare Improvement IHI; 2016 [Available from: www.ihi.org.
22. Barnett N, Athwal, D. & Rosenbloom, K. Medicines-related admissions: You can identify patients to stop that happening. Pharm J. 2011;286:471–2.
23. The National Health Service - West Midlands Strategic Health Authority. Risk indicators for medicines-related problems. 2012. (Available from: www.westmidlandsdeanery.nhs.uk/).
24. Holt S, Schmiedl S, Thurmann PA. Potentially inappropriate medications in the elderly: the PRISCUS list. Dtsch Arztebl Int. 2010;107(31-32):543-51.
25. Tommelein E, Mehuys E, Petrovic M, Somers A, Van Damme C, Pattyn E, et al. Potentially inappropriate prescribing in nursing home residents detected with the community pharmacist specific GheOP(3)S-tool. Int J Clin Pharm. 2016;38(5):1063-8.

Supplementary Material S5

Risk factors identified through literature review 2

The following tables contain the risk factors newly identified through the literature research, including a brief description of the respective literature.

Table S5.1: Risk factors identified by literature review

New factors included in the Delphi round 2 (Section 4) are written in italic.

Subject area	Risk factor	Number of mentions
Demographic factors	*Patients who speak foreign languages or belong to other cultures*	*1*
Social situation	Living alone	1
Age/health-related problems	Age (different cut-off values depending on the publication)	*3*
Age/health-related problems	Confusion states that have occurred	3
Age/health-related problems	*Frail patients /Frailty, sarcopenia, loss of functional abilities (physical "loss of strength and endur-ance", psychological "no desire to eat" and sociological components "loneliness"), slowing down/shortening of distances)*	*2*
Age/health-related problems	*Cardiovascular complications (occurred or potential)*	*2*
Age/health-related problems	Previous fall (3 months)	2
Age/health-related problems	Age-dependent physiological parameters such as kidney performance changed	2
Age/health-related problems	*Patients with chronic pain*	*1*
Social situation	Living alone	1
Healthcare	*Recent hospitalization (1 month; reasons: change in treatment plan, dosages, etc.)*	*2*
Healthcare	*Limited exchange of information/communication between doctor, nursing staff, patients*	*4*
Healthcare	*Several treatment plans at the same time from different providers*	*5*
Healthcare	*More than 1 prescriber, pharmacist*	*2*
Healthcare	*Many different healthcare professionals involved*	*2*

**Subject area**	**Risk factor**	**Number of mentions**
Diagnoses	Non-treated indications	5
Diagnoses	Co-morbidities	4
Diagnoses	Existing diagnosis of heart failure	3
Diagnoses	Multiple chronic diseases such as asthma, diabetes, hypertension, hypercholesterolemia, heart defects, dialysis, post-myocardial infarction, stroke, depression	3
Diagnoses	Cognitive deficits	3
Prescription	Increasing number of medications associated with increasing risk of MRPs (≥5, ≥8 , ≥10; different cut-off values depending on the publication)	13
Prescription	*Dose or strength of the medication not adjusted*	*9*
Prescription	Unnecessary or incorrect medication/therapy (suboptimal therapy: patient is not treated according to current guidelines or the therapy used does not achieve the desired effects)	9
Prescription	Side effects (suffered or potential)	9
Prescription	Duplications	3
Prescription	Medication - Psychotropic effect	2
Prescription	Drug-Drug Interaction	2
Prescription	*Medication without indication*	*1*
Prescription	Patients with high medication costs	1
Prescription	Previous medication error	1
Prescription	Frequent changes to treatment plans (number unclear)	1

**Subject area**	**Risk factor**	**Number of mentions**
Medication / Medication groups	Inappropriate Drugs, (geriatric drugs in general)	11
Medication / Medication groups	High-risk medications/medication groups (Proton pump inhibitors, inhalers, insulin, anticoagulants, ACE inhibitors, non-steroidal antiinfllammatory drugs, amlodipine, chemotherapeutics, immunosup-pressants, levodopa, lithium, opiates, digoxin, anticholinergics, benzodiazepines, diuretics, acetyl salicylic acid, folic acid, citalopram, simvastatin)	11
Medication / Medication groups	Drugs with influence on the nervous system, cardiovascular, metabolism, (non-steroidal anti-inflam-matory drugs)	3
Medication / Medication groups	Medication - Psychotropic effect	2
Medication / Medication groups	Over-the-counter medications	1
Medication Management	Poor adherence/compliance (partially incorrect intake due to not understanding the therapy)	10
Medication Management	*Timeliness/correctness/availability of medication lists*	*9*
Medication Management	*Lack of understanding of the therapy (often due to insufficient patient information)*	*2*
Medication Management	*Inadequate monitoring (irregular follow-up)*	*5*
Medication Management	Complex therapy plans	1
Medication Management	*Personal preferences not taken into account in the choice of medication*	*1*
Medication Management	*Patient or caregiver has concerns about medication or treatment plan*	*1*
Medication Management	Over-the-counter medications	1

MRP Medication related problem

Translated with the help of Deepl pro® (www.deepl.com, 31.12.2024)

Table S5.2: References of the risk factors identified by the literature search 2 (in alphabetical order)

	**References**
1	Atkinson, W. & Frey, D. Integration of a medication management model into outcome-based quality improvement: A pilot program in a rural proprietary home healthcare agency. Home Health Care Services Quarterly. 24, 29-45 (2008).
2	Boling, P. A. Strategic use of Home Care pharmacy consultation may be worthwhile. Journal of the American Geriatrics Society. 9, 1597-1598 (2002).
3	Brown, N. J., Griffin, M. R., Wayne, A., et al. A model for improving medication use in home health care patients. J. of the American Pharmaceut. Association, 38, 696–702 (1998).
4	Cannon, K. T., Choi, M. M. & Zuniga, M. A Potentially Inappropriate Medication Use in Elderly Patients Receiving Home Health Care: A Retrospective Data Analysis. Am J. Geriatr .Pharmacother. 4, 134-143 (2006).
5	Corsi, K., Lemay, V., Orr, K. K. & Cohen, L. Pharmacist medication therapy management in home health care: Investigation of a sustainable practice model. J. Am. Pharm. Assoc. 58, 64–68 (2018).
6	Devik, S. A., Olsen, R. M., Fiskvik, I. L., et al. Variations in drug-related problems detected by multidisciplinary teams in Norwegian nursing homes and home nursing care. Scandinavian Journal of Primary Health Care. 36, 291–299 (2018).
7	Elliott, R. A., Lee, C. Y., Beanland, C., et al. Development of a clinical pharmacy model within an Australian home nursing service using co-creation and participatory action research: the Visiting Pharmacist (ViP) study. *BMJ Open*. 7, (2017).
8	Foubert, K., Mehuys, E., Claes, L., et al. A shared medication scheme for community dwelling older patients with polypharmacy receiving home health care: role of the community pharmacist. Acta Clinica Belgica: International Journal of Clinical and Laboratory Medicine. Taylor and Francis Ltd. (2018).
9	Gomez, M. A., Villafaina, A., Hernandez, J., et al. Promoting appropriate drug use through the application of the spanish drug-related problem classification system in the primary care setting. Ann. Pharmacother. 43, 339-346 (2009).
10	Lee, C. Y., Beanland, C., Goeman, D., et al. Improving medication safety for home nursing clients: A prospective observational study of a novel clinical pharmacy service—The Visiting Pharmacist (ViP) study. J. of Clin. Pharm. and Ther. 43, 813–821 (2018).
11	Lee, C. Y., Goeman, D., Beanland, C., Elliott, R. A. Challenges and barriers associated with medication management for home nursing clients in Australia: a qualitative study combining the perspectives of community nurses, community pharmacists and GPs. *Fam Pract*. 1-11 (2018)
12	Lenander, C., Bondesson, A., Viberg, N., et al. Effects of medication reviews on use of potentially inappropriate medications in elderly patients; a cross-sectional study in Swedish primary care. BMC Health Services Research. 18, 616 (2018).

	References
13	Mahan, K. R., Clark, J., Anderson, K., et al. Development of a tool to identify problems related to medication adherence in home healthcare patients. Home Healthc. Now. 35, 277–282 (2017).
14	Meredith, S., Feldman, P. H., Frey, D., et al. Possible medication errors in home healthcare patients. J. Am. Geriatr. Soc. 49, 719–724 (2001).
15	Miettinen, M., Tiihonen, M., Hartikainen, S. & Nykänen, I. Prevalence and risk factors of frailty among Home Care clients. BMC Geriatrics. 17, 266 (2017).
16	Reidt, S., Morgan, J., Larson, T., & Blade, M. A. The Role of a Pharmacist on the Home Care Team: A collaborative model between a college of pharmacy and a Visiting Nurse Agency. Home Healthcare Nurse. 31, 80–87 (2013).
17	Snyder, M. E., Deshotels, D., Zilich, A. J., et al. A randomized, controlled pragmatic trial of telephonic medication therapy management to reduce hospitalization in home health patients. Health Services Research. 49, 1537–1554 (2014).
18	Toivo, T., Dimitrow, M., Puustinen, J., et al. Coordinating resources for prospective medication risk management of older Home Care clients in primary care: procedure development and RCT study design for demonstrating its effectiveness. BMC Geriatrics. 18, 74 (2018).
19	Triller, D. M., Clause, S. L., Briceland, L. L. & Hamilton, R. A. Resolution of drug-related problems in Home Care patients trough a pharmacy referral service. Am. J. Health-Syst. Pharm. 60, 905-910 (2003).
20	Vink, J., Morton, D. & Ferreri, S. Pharmacist identification of medication-related problems in the Home Care setting. The Consult. Pharma. 26, 477-484 (2011).
21	Wellman, B., Frail, C., Zillich, A. & Snyder, M. Pharmacists' experiences with a telephonic medication therapy management program for home health care patients, Consult. Pharma. 30, 163-170 (2015).

Table S5.3: Summary of the studies included in literature review 2

	Author<br>Year	Title	Subject	Results<br>Conclusions	Remarks<br>(by the research team)
1	Atkinson et al.<br>2008	Integration of a Medication Management Model into Outcome-Based Quality Improvement: A Pilot Program in a Rural Proprietary Home Healthcare Agency	The article describes a successful initiative to involve a pharmacist. Centered medication management program in the home care sector. Use OASIS (high risk patients detect)	Geriatric Medication Assessment Protocol (GMAP) to identify high-risk patients as a first step and subsequent pharmaceutical clarification. In addition, a medication database and patient indicators are screened to identify high-risk patients.	Home care setting given, as well as population elderly patients; has many other studies that would be suitable as references
2	Boling et al.<br>2002	Strategic use of Home Care pharmacy consultation may be worthwhile.	Pharmaceutical interventions are bearing fruit in that the prescription of medication has changed.	It lists MRPs and discusses which problems are particularly relevant in the home care setting. It also questions the political stance and the problem of why pharmacists are not supported in the home care setting, even though clear benefits of pharmaceutical interventions have been demonstrated in other settings.	Setting is right and good food for thought and argumentation on the political stance towards home care settings and pharmaceutical interventions
3	Brown et al.<br>1998	A model for improving medication use in home health care patients	Development of a similar tool	Only the development has been described, but the tool has not yet been validated	No concrete MRPs from experience of using interventions, therefore only good as background information, but not for answering literature questions; setting is correct
4	Cannon et al.<br>2006	Potentially inappropriate medication use in elderly patients receiving home health care: a retrospective data analysis	Medication management often focuses on the hospital setting; this study examines the impact in home health care (HHC)	Identification of the prevalence of PIM, dangerous drug interactions and other patterns of medication use. PIM and DDI are prevalent and polypharmacy is associated with higher rates of PIM use and DDIS	Home care setting and population are right.
5	Corsi et al.<br>2018	Pharmacist medication therapy management in home health care: Investigation of a sustainable practice model	Aim of the study financial benefits of interventions and identification of types of MRPs in the home health care population.	Risk factors were mentioned, as were the frequently occurring MRPs	Setting matches nonprofit home health care agency, age 65 and older
6	Devik et al.<br>2018	Variations in drug-related problems detected by multidisciplinary teams in Norwegian nursing homes and home nursing care.	The aim of the study is to find factors tailored to the home care setting in addition to the number of medications as a risk factor. To describe and compare the MRPs with the MRPs in nursing homes	Good enumeration of MRPs and pointing out differences to nursing homes.	Setting is right, but combined/compared with nursing homes.
7	Elliott et al.<br>2017	Medicines management, medication errors and adverse medication events in older people referred to a community nursing service: a retrospective observational study	Collaboration between nurses, pharmacists and patients should be developed.	Data and analysis of given reflections and feedback from meetings and interviews conducted by clinical pharmacists were used as a basis. Direct and indirect care by pharmacists (clear assignment of tasks). Interdisciplinary collaboration has been successful and should be recognized nationally and internationally.	Non profit home nursing service in Australien; Setting is right.

	Author Year	Title	Subject	Results Conclusions	Remarks (by the research team)
8	Foubert et al. 2018	A shared medication scheme for community dwelling older patients with polypharmacy receiving home health care: role of the community pharmacist	Study was done with community pharmacists and "not yet in" multidisciplinary team.	Objective the differences/changes from pharmaceutical to nursing medication programs and possible improvements through the contribution of a pharmacist to the medication programs in a multidisciplinary collaboration	Home care setting given, population over 70 years of age; pharmacist is not included as a health care professional in the interdisciplinary team, but is the "public" pharmacist, but in collaboration with nurse and doctor
9	Gomez et al. 2009	Promoting appropriate drug use through the application of the Spanish drug-related problem classification system in the primary care setting	MRP classification system in primary care setting	is only not exactly defined in the outpatient setting! The tool is helpful for systematically detecting and documenting MRPs (daily general practice).	Primary care setting and drug-related problem classification system; **not** called home care setting but health care centers? No nurses named as caregivers.
10	Lee et al. 2018	Improving medication safety for home nursing clients: A prospective observational study of a novel clinical pharmacy service-The Visiting Pharmacist (ViP) study	The aim of the study is to find the number and type of medication-related problems (MRPs) and discrepancies in medication/therapy	Patients are screened by nurses for risk situations and, if necessary, patients are referred to clinical pharmacists, who then make home visits and reconcile medication and carry out a detailed medication review; the current medication list and suggestions for optimization are forwarded to GPs, nurses, etc.	Setting is right, and MRPs are discussed. The model and risk tool are very similar to those envisaged in this framework.
11	Lee et al. 2018	Challenges and barriers associated with medication management for home nursing clients in australia: a quantitative study	Study: What influence an interdisciplinary team can have on the medication process in the home-nursing setting	Describes barriers and challenges in medication management; interviews and meetings to optimize, create clear divisions of labor and reduce ineffective teams. Strategies were developed to improve interdisciplinary medication management and medication safety in home nursing settings.	Home care setting and population are right.
12	Lenander et al. 2018	effects of medication reviews on use of potentially inappropriate medications in elderly patients; a cross-sectional study in swedish primary care	Study to evaluate the medication reviews and recognize the PIMs responsible for many MRPs.	After the intervention and the study, the prescription of PIMs, especially psychotropics, decreased. MRPs that occurred are described.	over 75 but both settings Nursing homes and home care patients
13	Mahan et al. 2017	Development of a Tool to Identify Problems Related to Medication Adherence in Home Healthcare Patients	A similar risk tool that has been tested, a list of questions to detect people at risk of MRPs in the home care setting	The questionnaire was tested and optimized. In order to detect patients at risk of medication-related nonadherence more quickly and specifically. Non-profit agency in collaboration with the university.	Home care setting and population match; risk tool is tested and optimized
14	Meredith et al. 2001	Possible medication errors in home healthcare patients	To determine the number of possible medication errors in this population	Risk factors were found and described	Home health-care patients over 65, setting is right

	Author<br>Year	Title	Subject	Results<br>Conclusions	Remarks<br>(by the research team)
15	Miettinen et al.<br>2017	Prevalence and risk factors of frailty among home care clients	Early recognition of frailty is important to prevent problems later on.	effects of medication reviews on use of potentially inappropriate medications in elderly patients; a cross-sectional study in swedish primary care	Home care clients and population over 75 years: But the focus is not on pharmacological therapy but on frailty, which also has an effect on falls and the physiology of patients in old age.
16	Reidt et al.<br>2013	The role of a pharmacist on the home care team: a collaborative model between a college of pharmacy and a visiting nurse agency	MRPs are common in the home care setting. The non-profit organization described consists of nurses and physiotherapists.	Pharmaceutical services are described and also that some agencies already integrate pharmacists. The agency discovered the need to call in an "expert" for complex medications, resulting in a collaboration with the university. The whole process of pharmaceutical home visits is explained.	Home care setting; pharmacy students are employed.
17	Snyder et al.<br>2014	A Randomized, Controlled Pragmatic Trial of Telephonic Medication Therapy Management to Reduce Hospitalization in Home Health Patients	The effectiveness of telephone medication management (MTM) in home care patients is being tested. The endpoint was the potential of MTM to reduce unnecessary hospitalizations	Risks for hospitalization, number of medications and other OASIS-C (99 risk factors) data elements are considered. MTM did not show the same benefit in all patients, the patients with the lowest risk profile were most likely to benefit from MTM and did not need to be hospitalized within 60 days.	Home care setting is given; but other method using telephone medication management by a pharmacist (MTM various telephone appointments)
18	Toivo et al.<br>2018	Coordinating resources for prospective medication risk management of older home care clients in primary care: procedure development and RCT study design for demonstrating is effeciveness	coordinated medication management model (CoMM) with 5 stages is being tested for effectiveness in an RCT.	Interesting approach to avoid MRPs with different tools and collaboration between nurses and pharmacists. Tools and collaboration between nurses and pharmacists. Clear division of tasks among healthcare professionals, with nurses primarily involved in initial screening.	Home care setting is given and population is also right
19	Triller et al.<br>2003	Resolution of drug-related problems in home care patients trough a pharmacy referral service	A model designed to detect high-risk patients	A medication management program for the identification and categorization of MRPs in home health care (HCC) was investigated	Home care setting and pharmaceutical interventions, important source
20	Vink et al.<br>2011	Pharmacist identification of medication-related problems in the Home Care setting	Identify medication-related problems in home care patients.	One of the objectives was to describe the likelihood of occurrence and type of MRP's that were not recognized in a previous medication review conducted by other healthcare professionals. The review process identified the main problems of MRP's with suboptimal therapy and unnecessary medications. Recommendations were often to stop the medication or recommend consultation with a doctor.	Home care patient, nonprofit agency; Setting given; Only patients considered who need to take **eight or more** medications.
21	Wellman et al.<br>2015	Pharmacists' experiences with a telephonic medication therapy management program for home health care patients	Understanding perceived barriers and facilitators of MTM/ Telephone MTM for home health care patients	Strategies for optimizing MTM in the home care setting; pharmaceutical interventions and observations are discussed	Home care setting and pharmaceutical interventions

Abbreviation: MRP Medication related problem

Translated with the help of Deepl pro® (www.deepl.com, 31.12.2024)

Supplementary Material S6: Form Delphi round 2 with all risk factors

Name, first name *(please overwrite)*

Section 1: Risk factors of the created risk tool (choice of risk factors: Delphi round one 2018).

		suitable	Nominations Literature	Sources	Comments
	**Patient - social situation**				
	What social situation do you consider a risk factor for DRPs?				
S4	Communication problems - foreign language		2	15,17	
	**Healthcare**				
	Which of the following issues do you consider a risk factor for DRPs?				
**H4 so far**	Frequent change of doctor	☐	1	21	
**H4 new**	Multiple changes of physician within a calendar year or multiple prescribing physicians at the same time (without the knowledge of the doctors involved)	☐	Optimization suggestion		
**H11 so far**	Transition from hospital to Spitex (especially after hospital admission due to DRPs).	☐	1	22	
**H11 new**	Rehospitalizations or multiple transfers and passing through various interfaces	☐	Optimization suggestion		
	**Diagnoses**				
	What diagnoses do you consider to be at risk for DRPs?				
DIA7	Renal dysfunction GFR <30ml/min		2	7, 17	
	**Age/health-related problems**				
	Which of the following issues do you consider a risk to DRPs?				
AG5	cognitive deficits		5	1, 9, 14, 15, 22	
	**Prescription**				
	What number of prescribed medications/medication changes do you consider to be at risk for DRPs?				
P3	Number of drugs ≥7		1	4	
	What medications/groups of medications do you consider to be at risk for DRPs?				
**M1 so far**	Drugs with narrow therapeutic range	☐	8	3, 4, 6, 9, 12, 15, 24, 25	
**M1 new**	Drugs with narrow therapeutic range (list of drugs) (Which drugs do you consider particularly sensitive? **Please give examples in the orange highlighted backmost column "Remarks"**).	☐	Optimization suggestion		
M3	Medications that are unsuitable for geriatric patients (e.g.Priscus).		>10	13, 24	
M9	Psychotropic drugs ≥3 (centrally acting analgesics, antipsychotics, antidepressants, benzodiazepines).		1	2	
	**Medication Management**				
	Which of the following situations do you consider a risk to DRPs?				
MM1	Patient does not have a current medication list		1	4	
**MM5 so far**	Patient has difficulty distinguishing the tablets, also visual difficulties	☐	2	15,22	
**MM5 new**	Patient has difficulty distinguishing the tablets from each other, especially with new-onset visual impairments	☐	Optimization suggestion		
MM10	Patient takes medication without doctor's knowledge (also self-purchased)		5	4, 10, 11, 15, 25	
MM12	Patient - lack of understanding of therapy and disease		4	4, 10, 11, 15	

Optimization - risk assessment - medication-associated problems (DRPs) in Spitex patients.

0=no risk; 1=minimal risk; 2=small risk; 3=medium risk; 4=increased risk; 5=considerable risk; 6=large risk

Section 2: Scarcely failed risk factors (choice of risk factors: 1st Delphi round 2018).

		Risk	Nominations Literature	Sources	Comments
	**Patient - social situation**				
	What social situation do you consider a risk factor for DRPs?				
S5	Communication problems - Hearing problems		*new*		
	**Healthcare**				
	Which of the following issues do you consider a risk factor for DRPs?				
H10	≥4 emergency/hospital admissions or >3 rehospitalizations within the past 12 months.		1	17	
	**Prescription**				
	What number of prescribed medications/medication changes do you consider to be at risk for DRPs?				
P6	≥5 medication changes within the last 12 months.		1	5	
	What medications/groups of medications do you consider to be at risk for DRPs?				
M4	Duplicate prescription of a class of drugs		1	13	
M5	Anticoagulants		27	3, 4, 6, 9, 14, 15, 17, 20, 22, 23, 24, 25	
M10	Benzodiazepines / Z-Drugs		41	2, 6, 13, 24, 25	
M12	Anticholinergics		11	1, 6, 15, 24	
M17	Antidiabetics - Insulin		11	3, 5, 6, 9, 14, 15, 17, 19, 22, 23	
M18	Metothrexate		5	3, 4, 20, 22, 23	
	**Medication Management**				
	Which of the following situations do you consider a risk to DRPs?				
MM2	poor comprehensibility of the therapy plan (complex therapy plans)		2	11, 22	
MM6	Patient has difficulty swallowing the tablets		5	11, 14, 15, 22, 23	
MM7	Chaotic drug storage		*new*		
MM9	Non-adherence/lack of compliance (tw. incorrect intake due to not understanding the therapy)		7	4, 10, 15, 17, 18, 22, 25	
MM11	Patient takes medication differently than prescribed by the doctor		5	4, 8, 10, 11, 22	
MM13	Concerns about medication - patient		5	4, 8, 10, 11, 15	
MM16	Adverse drug reaction(s) in the past		2	16, 23	

Section 3: New risk factors (by experts of the Delphi round one 2018)

		Risk	Nominations Literature	Sources	Comments
	**Patient - demographic data**				
	What age do you consider a risk factor for DRPs?				
D3	≥50 years		new		
D4	≥65 years & multimorbidity & polypharmacy.		new		
D5	≥75 years & multimorbidity & polypharmacy.		new		
D6	≥80 years		new		
	**Patient - social situation**				
	What social situation do you consider a risk factor for DRPs?				
S6	No relatives in the vicinity		*new*		
S7	Person in need of care in the household (disturbance, excessive demand)		*new*		
S8	Homebound in combination with cognitive problems or lack of social support.		*new*		
S9	financial difficulties (deductible)		*new*		
S10	Educational level low		*new*		
	**Healthcare**				
	Which of the following issues do you consider a risk factor for DRPs?				
H15	changing prescribers (hospital, spa, general practitioner, specialist)		*new*		
H16	inadequate assessment of own competencies regarding medication management		*new*		
H17	No internal contact person after discharge from the hospital		*new*		
H18	No available family doctor		*new*		
H19	Communication problems with the family doctor		*new*		
	**Diagnoses**				
	What diagnoses do you consider to be at risk for DRPs?				
DIA13	neurocognitive disorders of all severities		*new*		
DIA14	≥4 chronic diseases		*new*		
DIA15	Liver failure		*new*		
DIA16	mental illnesses		*new*		
	**Age/health-related problems**				
	Which of the following issues do you consider a risk to DRPs?				
AG6	Visual impairment, visual disturbances especially if new in old age and medication management is done independently		*new*		
AG7	Polyarthritis		*new*		
AG8	Addictive disorders involving substance abuse/daily use of addictive substances (such as alcohol, benzodiazepines, cocaine, heroin	in,)	*new*		

<u>Section 3 *cont.* :</u> New risk factors (by experts of the 1st Delphi round 2018)

		Risk	Nominations Literature	Sources	Comments
	**Prescription**				
	What number of <u>prescribed medications/medication changes</u> do you consider to be at risk for DRPs?				
P8	Multiple switching between different generics		*new*		
P9	Number of drugs ≥10 (already included in the previous risk tool Number of drugs ≥7).		*new*		
	What situation related to <u>interactions</u> do you consider a risk for DRPs?				
P10	Clinically relevant interaction without possible monitoring		*new*		
	What <u>medications/groups of medications</u> do you consider to be at risk for DRPs?				
M19	Digoxin		*new*		
M20	Lithium		*new*		
M21	Neuroleptics		*new*		
M22	strong CYP and p-Gp inducers/inhibitors		*new*		
M23	duplicate prescription of a substance (generic and original)		*new*		
	**Medication Management**				
	Which of the following situations do you consider a risk to DRPs?				
MM17	Patient has no/lack of insight into the illness or lack of insight into the need for help.		*new*		
MM18	Therapy goal does not correspond to the client goal		*new*		
MM19	Many interfaces from the prescription to the client taking the medication		*new*		

Section 4: New risk factors from the literature review two, 2019.

		Risk	Nominations Literature	Sources	Comments
	**Patient - demographic data**				
	What age do you consider a risk factor for DRPs?				
D7	Frail patients/Frailty* (definition includes the following characteristics: sarcopenia, loss of functional Abilities (physical "loss of strength and endurance", psychological "no desire to eat") and sociological components ("loneliness"), slowing/shortening of travel distances).		2	A6), A12), *F1)	
	**Patient - social situation**				
	What social situation do you consider a risk factor for DRPs?				
S11	Patients speaking foreign languages or belonging to other cultures		2	A11)	
	**Healthcare**				
	Which of the following issues do you consider a risk factor for DRPs?				
H20	recent hospitalization (1 month) (reasons: change in therapy schedule, dosage, etc.)		2	A6), C1)	
H21	limited exchange of information/communication between doctor, nursing, patients		4	A6), A7), A11), D4)	
H22	Patients with high medication costs		1	A4)	
H23	Multiple medication lists at the same time, especially when several prescribers (various specialists involved) are involved.		5	A6), A11), B2), B3), B4)	
H24	several different "health-care-professionals" involved		2	A2), A3)	
	**Diagnoses**				
	What diagnoses do you consider to be at risk for DRPs?				
DIA17	present diagnosis of heart failure		3	A4), D1), D2)	
	**Age/health-related problems**				
	Which of the following issues do you consider a risk to DRPs?				
AG9	cardiovascular complications (occurred or potential)		2	A4), D3)	
AG10	occurred states of confusion		3	A4), D2), D3)	
AG11	Patients with chronic pain		1	A4)	
AG12	Indications not treated		5	A1), B4), D1), D2), D4)	
	**Prescription**				
	What situation related to interactions do you consider a risk for DRPs?				
P11	Dose or strength of the drug not adjusted		9	A1), A3), A4), A12), B2), B4), D1), D2), D4)	
P12	Medication without indication		1	A3)	
P13	medication that is not necessary or incorrect therapy (suboptimal therapy: patient is not treated according to current Guidelines treated or the applied therapy does not achieve the desired effects).		9	A4), A5), A12), B2), B4), D1), D2), D3), D4	

	**Section 4 *cont.* : New risk factors from the literature search (2019)**	**Risk**	**Nominations Literature**	**Sources**	**Comments**
	What medications/groups of medications do you consider to be at risk for DRPs?				
M24	High-risk drugs/groups of drugs<br>(Which medications do you consider particularly risky?<br>**Please give examples in the green highlighted backmost column "Remarks"**)		11	(A2), (A3), (A6), (A7), (A8),<br>(A9), (A12), (B2), (B3), (D3),<br>D4)	

	**Medication Management**				
	Which of the following situations do you consider a risk to DRPs?				
MM20	Lack of understanding of the therapy (often due to inadequate patient information)		2	A3), A11)	
MM21	Personal prevalences not taken into account in the choice of medication		1	A3)	
MM22	Lack of timeliness/correctness/lack of drug lists.		9	A2), A3), A6), A9), A10), A11), B2), B4), D4)	
MM23	inadequate monitoring (irregular follow-up)		5	A2), A4), A5), D1), D2)	
MM24	Patient and/or caregiver have concerns regarding medications or treatment plan		*1*	A11)	

	**Favorites of the risk factors (from blocks 2-4 of the categories to be evaluated).**
	Please list a MAXIMUM of 10 influencing factors that you consider to be key cues for subsequent DRPs:
1	
2	
3	
4	
5	
6	
7	
8	
9	
10	
